# Penetration of the Stigma and Style Elicits a Novel Transcriptome in Pollen Tubes, Pointing to Genes Critical for Growth in a Pistil

**DOI:** 10.1371/journal.pgen.1000621

**Published:** 2009-08-28

**Authors:** Yuan Qin, Alexander R. Leydon, Ann Manziello, Ritu Pandey, David Mount, Stojan Denic, Bane Vasic, Mark A. Johnson, Ravishankar Palanivelu

**Affiliations:** 1 Department of Plant Sciences, University of Arizona, Tucson, Arizona, United States of America; 2 Department of Molecular Biology, Cell Biology, and Biochemistry, Brown University, Providence, Rhode Island, United States of America; 3 Arizona Cancer Center and Southwest Environmental Health Sciences Center, University of Arizona, Tucson, Arizona, United States of America; 4 Department of Electrical and Computer Engineering, University of Arizona, Tucson, Arizona, United States of America; Stanford University School of Medicine, United States of America

## Abstract

Pollen tubes extend through pistil tissues and are guided to ovules where they release sperm for fertilization. Although pollen tubes can germinate and elongate in a synthetic medium, their trajectory is random and their growth rates are slower compared to growth in pistil tissues. Furthermore, interaction with the pistil renders pollen tubes competent to respond to guidance cues secreted by specialized cells within the ovule. The molecular basis for this potentiation of the pollen tube by the pistil remains uncharacterized. Using microarray analysis in *Arabidopsis*, we show that pollen tubes that have grown through stigma and style tissues of a pistil have a distinct gene expression profile and express a substantially larger fraction of the *Arabidopsis* genome than pollen grains or pollen tubes grown *in vitro*. Genes involved in signal transduction, transcription, and pollen tube growth are overrepresented in the subset of the *Arabidopsis* genome that is enriched in pistil-interacted pollen tubes, suggesting the possibility of a regulatory network that orchestrates gene expression as pollen tubes migrate through the pistil. Reverse genetic analysis of genes induced during pollen tube growth identified seven that had not previously been implicated in pollen tube growth. Two genes are required for pollen tube navigation through the pistil, and five genes are required for optimal pollen tube elongation *in vitro*. Our studies form the foundation for functional genomic analysis of the interactions between the pollen tube and the pistil, which is an excellent system for elucidation of novel modes of cell–cell interaction.

## Author Summary

For successful reproduction in flowering plants, a single-celled pollen tube must rapidly extend through female pistil tissue, locate female gametes, and deliver sperm. Pollen tubes undergo a dramatic transformation while growing in the pistil; they grow faster compared to tubes grown *in vitro* and become competent to perceive and respond to navigation cues secreted by the pistil. The genes expressed by pollen tubes in response to growth in the pistil have not been characterized. We used a surgical procedure to obtain large quantities of uncontaminated pollen tubes that grew through the pistil and defined their transcriptome by microarray analysis. Importantly, we identify a set of genes that are specifically expressed in pollen tubes in response to their growth in the pistil and are not expressed during other stages of pollen or plant development. We analyzed mutants in 33 pollen tube–expressed genes using a sensitive series of pollen function assays and demonstrate that seven of these genes are critical for pollen tube growth; two specifically disrupt growth in the pistil. By identifying pollen tube genes induced by the pistil and describing a mutant analysis scheme to understand their function, we lay the foundation for functional genomic analysis of pollen–pistil interactions.

## Introduction

Cell-cell interactions can regulate the fate, morphology, and migration patterns of cells during development of multicellular organisms. Cell surface molecules mediate these interactions by initiating intracellular signal transduction cascades that cause changes in nuclear gene expression patterns [1]. Since the pollen tube of flowering plants interacts with several distinct cell types during its migration to an ovule, it represents an attractive model system for studying changes in global gene expression patterns in response to cell-cell interactions.

Flowering plants alternate between haploid gametophytic and diploid sporophytic phases of their life cycle. Male and female gametophytes develop through a series of mitotic divisions of haploid spores, which are produced when diploid sporophytic cells within the anther (male) and ovule (female) undergo meiosis [2]. Male spores divide asymmetrically to produce a vegetative cell that engulfs a smaller generative cell. The generative cell then divides to form two sperm cells within the cytoplasm of the pollen grain, which constitutes the mature male gametophyte [3],[4].

Upon binding a compatible stigma, the pollen grain germinates a tube that penetrates the stigma and grows rapidly through a protein and carbohydrate-rich extracellular matrix secreted by specialized cells of the pistil [5]. Pollen tubes extend by an actin-myosin-based tip-growth mechanism that transports vesicles loaded with new cell wall material to the extending apex [6]–[9]. In response to guidance cues from female cells, individual pollen tubes target and enter an ovule micropyle [10], contact the female gametophyte [11], arrest growth [12],[13], and burst [14], releasing two sperm for fertilization of female gametes [15].

Pollen is released from anthers at anthesis and has therefore been amenable to global gene expression profiling. Transcriptome analysis showed that pollen expresses a unique subset of the *Arabidopsis* genome relative to sporophytic tissues [16]–[19] and revealed changes in the patterns of gene expression as the male gametophyte develops from a spore to a tricellular pollen grain [18]. Determination of the transcriptome of purified sperm cells showed that male gametes have a distinct gene expression program that contributes to the transcriptome of the pollen grain [20]. Recently, genome-wide expression profiling of pollen tubes grown *in vitro* identified a set of genes that are expressed in the pollen tube but not in pollen [21]. This important study suggests that there is *de novo* mRNA synthesis in the growing pollen tube and raises the interesting possibility that a novel set of genes may be expressed in response to growth through the pistil.

Studies in maize and petunia suggest that pistils induce gene expression changes in pollen tubes. For example, exposure of petunia pollen to kaempferol, a pollen germination-inducing molecule produced by the stigma [22], resulted in significant gene expression changes during the first 0.5 hours after pollen germination. Eight novel cDNAs whose expression increased in response to kaempferol were identified in petunia pollen tubes [23].

It is also clear that pollen tube physiology changes as a consequence of growth through pistil tissue, but the molecular bases for these changes are largely unknown. Pollen tubes extend at faster rates in a pistil and achieve substantially greater terminal lengths compared to pollen tubes grown *in vitro* [24]. Furthermore, pollen tubes germinated *in vitro* target the ovule micropyle at very low efficiencies; however, if pollen tubes are first grown through pistil tissues, guidance to ovules is significantly enhanced [25],[26]. Therefore, it is likely that the transcriptome of pollen tubes grown through the pistil differs considerably from that of *in vitro*-grown pollen tubes. Defining these differences could lead to the discovery of genes that are activated by potentiation of the pollen tube by the pistil and are required for pollen tube guidance, and to the identification of gene regulatory networks that mediate the pollen tube response to the pistil environment.

In this study, we defined the transcriptome of pollen tubes that have grown through pistil tissues using a semi-*in vivo* pollen tube (SIV PT) growth system we developed for *Arabidopsis* [26]. Importantly, the SIV PT transcriptome was significantly different from those of pollen grains or pollen tubes grown *in vitro*. In addition, we showed that a significant number of genes are shared between the SIV PT transcriptome and sporophytic tissues, which are not expressed in pollen or pollen tubes grown *in vitro*. We also defined a set of genes that are enriched in semi-*in vivo* grown pollen tubes relative to pollen, pollen tubes grown *in vitro*, and a collection of sporophytic tissues. The distribution of functional categories in this set of genes compared to pollen grains revealed a significant enrichment for the Toll/Interleukin-1 Receptor homology-Nucleotide Binding Site-Leucine Rich Repeat (TIR-NBS-LRR)-type receptor family of proteins [27]. These genes have been implicated in pathogen-derived-effector-protein recognition and could play a direct signaling role during pollen tube potentiation by pistils. To determine whether genes whose expression increases during pollen tube growth *in vitro* and/or semi *in vivo* are required for pollen tube function, we performed reverse-genetic analysis of selected genes. We identified five mutants that disrupt pollen tube growth *in vitro* and two mutants that specifically disrupt pollen tube growth in the pistil. Our studies lay the foundation for functional genomic analysis of pollen tube-pistil interactions.

## Results

### Microarray Analysis To Identify Genes Expressed in *In Vitro*– and Semi *In Vivo*–Grown Pollen Tubes

**Figure 1:**
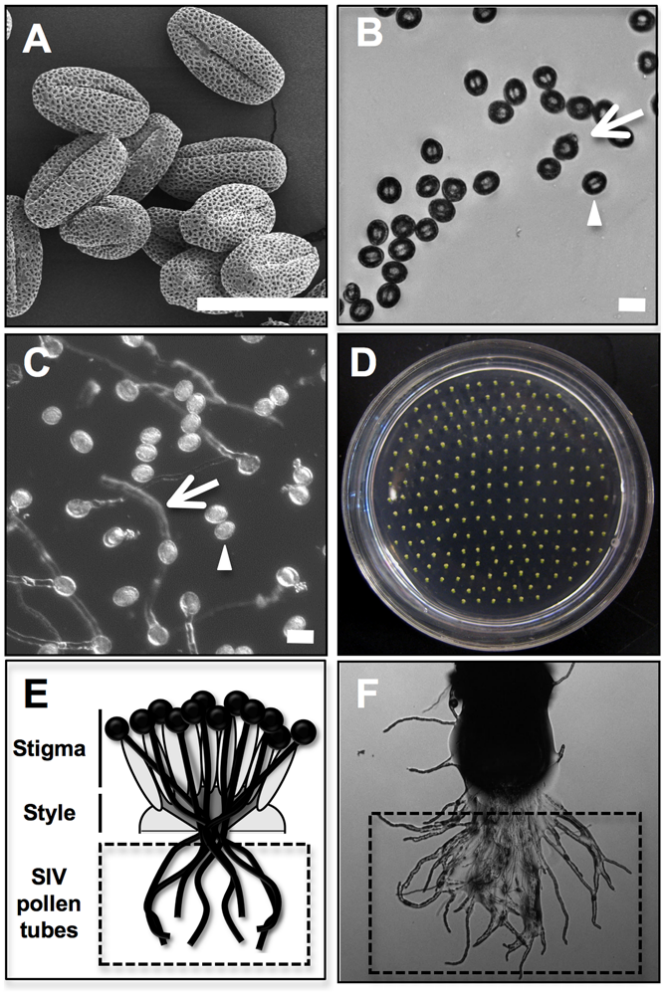
Pollen samples used in microarray analysis. (A) A scanning electron micrograph of dry pollen grains collected by vacuum. (B) Bright field micrograph of the 0.5 h PT sample. Hydrated pollen grains (a representative grain shown by arrowhead) and grains that are beginning to germinate (arrow) are shown. (C) Pollen tube growth in a 4 h PT sample. Many grains exhibit elongated tubes (arrow); however, some pollen grains have not germinated (arrowhead). (D) Light micrograph of a petri dish containing semi *in vivo* grown pollen tubes from cut pistil explants. Approximately 800 pollen tube bundles were used for each microarray experiment. (E) Diagram of semi *in vivo* pollen tube growth. Bundles of pollen tubes that have emerged from the style (within the dotted box) were excised for RNA isolation. (F) A higher magnification of one cut pistil explant is shown. The dotted box indicates the SIV PT material collected for microarray analysis. Scale bars (A–C), 25 µm.

To identify gene expression changes during pollen tube growth *in vitro* or through a pistil, we performed comparative microarray analysis with RNA isolated from dry, un-germinated pollen (dry pollen, Figure 1A), pollen grown *in vitro* for 0.5 hours (0.5 h PT, Figure 1B), or for 4 hours (4 h PT, Figure 1C) and pollen germinated and grown through the stigma and style (SIV PT; Figure 1D-1F). Pollen tubes grown by the semi-*in vivo* method exit as a bundle from the cut end of a style and fan out on the solid pollen growth medium (Figure 1E, 1F). Pollen tube bundles from ∼800 cut pistils were excised and combined for RNA isolation (Figure 1E, 1F).

Using RNA isolated from the four different pollen conditions (dry pollen, 0.5 h PT, 4 h PT and SIV PT; also see Materials and Methods), we synthesized probes and hybridized to Affymetrix *Arabidopsis* ATH1 genome arrays. We generated probes from four biological replicates of dry pollen, 0.5 h PT, 4 h PT and three replicates of SIV PT. The raw expression data from these 15 experiments are provided (Table S1, Table S2, Table S3, Table S4). To detect genes that are preferentially expressed in pollen tubes compared to other cell types, we obtained publicly available microarray data for seven sporophytic tissues: 7-day-old roots, 17-day-old roots, 8-day-old seedlings, 21-day-old seedlings, 17-day-old rosette leaves (three replicates of each, [28]), unpollinated ovary and unpollinated stigma (four and three replicates respectively, [29]). Data from a previously published pollen microarray was also included as a reference (three replicates, [28]). By analyzing these 25 publicly available data sets along with our 15 arrays (Robust Microarray Analysis tools, RMA, see Materials and Methods), we obtained normalized expression values for each gene that allowed us to make comparisons among these experiments (Table S5).

The ranges of Pearson coefficients of array-array intensity were high for pairwise comparisons among the dry pollen, 0.5 h PT, 4 h PT and SIV PT replicates, suggesting that there is high reproducibility among the biological replicates and that one pollen type could be distinguished from the other (Table S6). Similar results were obtained with hierarchical clustering of pollen arrays (Figure S1). Pairwise comparisons of Pearson correlation coefficients showed that previously published pollen data [28] was most similar to our dry pollen (0.935–0.944) and 0.5 h PT (0.935–0.949) samples.

### Characterization of the *Arabidopsis* Pollen Tube Transcriptome

**Figure 2:**
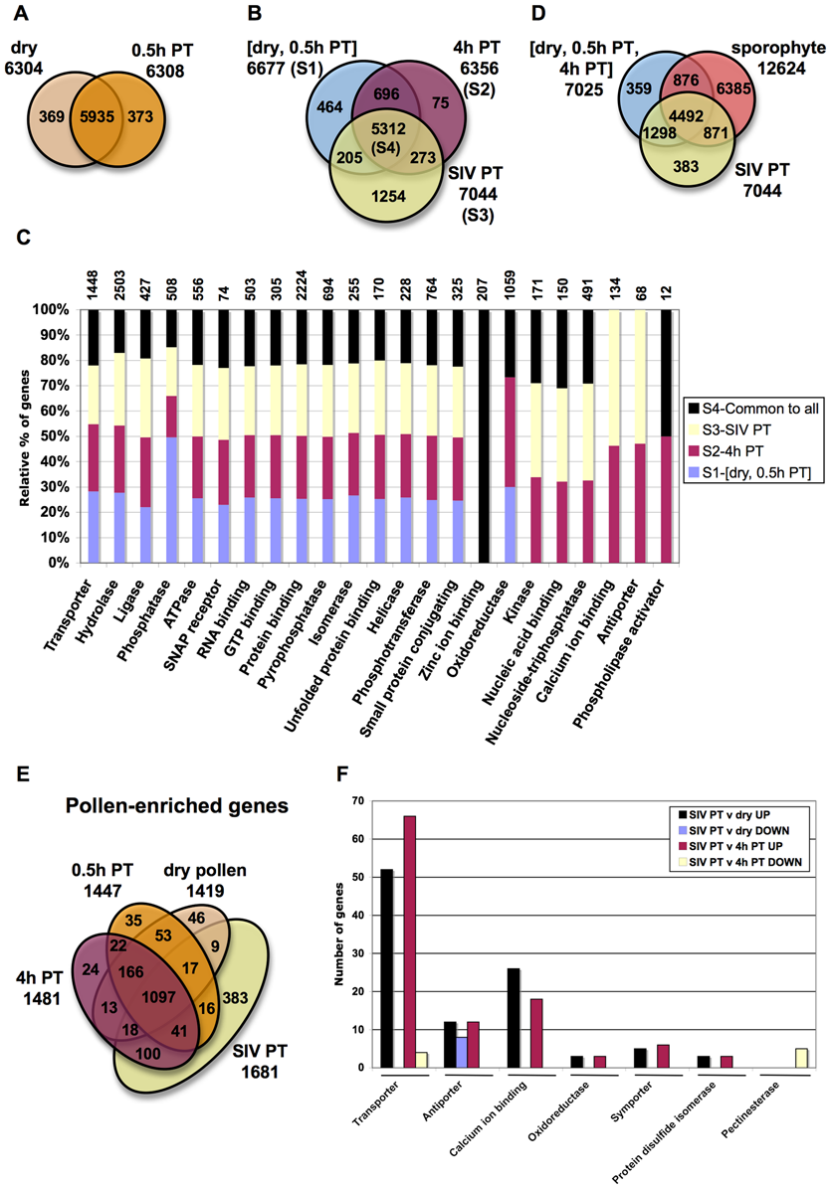
Differential gene expression in pollen and pollen tubes. Genes were considered expressed in a given cell type or tissue if their mean, normalized expression value was greater than 100; the total number of genes expressed in each category is provided. (A) A two-way comparison of dry pollen (dry) with 0.5 h PT. (B) The set of 6,677 genes expressed in dry and 0.5 h PT in a 3-way comparison with 4 h PT and SIV PT. (C) Relative percentage of genes with selected GO terms that were significantly overrepresented in sectors 1–4 in Figure 2B. The number above each column denotes the cumulative number of genes detected for a particular GO term in the four sectors (also see Materials and Methods). (D) The set of 7,025 genes expressed in dry, 0.5 h PT, and 4 h PT in a 3-way comparison with the set of genes expressed in any of the seven sporophytic tissues analyzed (sporophyte) and SIV PT. (E) A 4-way comparison among the pollen samples of 2,040 pollen-enriched genes that were not expressed in any of the seven sporophytic tissues analyzed. (F) Number of genes with selected GO terms that were significantly overrepresented in the genes that are up or down regulated significantly in SIV PT compared to dry pollen or 4 h PT (Table S14 and Table S15).

Using our RMA-normalized data set (Table S5), we identified genes that are expressed during pollen tube growth. RMA analysis does not provide a ‘present’ or ‘absent’ score, so we set an expression value of 100 or higher as a stringent threshold for expression to obtain a conservative estimate of gene expression in each cell or tissue analyzed. In reverse transcription followed by real-time quantitative PCR (qRT-PCR) experiments, we could consistently confirm expression for genes above this threshold value (see below). Using these criteria, we found that 6,304, 6,308, and 6,356 genes were present in dry pollen, 0.5 h PT, and 4 h PT, respectively (Figure 2A, 2B). The number of genes expressed in SIV PT was greater (7,044) than any other pollen tube growth condition tested, suggesting a substantial change in the transcriptome following interaction with female reproductive tissues (Figure 2B).

We determined the extent of overlap among the transcriptomes of the four pollen conditions we tested. There is significant overlap between dry pollen and 0.5 h PT (Figure 2A; Table S6, Table S7, and Figure S1). Because of this extensive similarity, we combined dry pollen and 0.5 h PT into one group of 6,677 pollen genes [dry pollen and 0.5 h PT] (sector S1, Figure 2B). This combined set, when compared with 4 h PT (sector S2, Figure 2B) and SIV PT (sector S3, Figure 2B) identified 5,312 genes (sector S4, Figure 2B) shared by all pollen samples, representing a core set of pollen genes. Previously characterized pollen and pollen tube-expressed genes known to be critical for pollen tube growth such as the *ROP1* GTPase (At3g51300, [30]), *AtGEF12* (At1g79860, [31]), *RabA4d* (At3g12160, [32]), *ACA9* (At3g21180, [33]), *CNGC18* (At5g14870, [34]), the *VANGUARD* pectinesterase (At3g621790, [35]), and *AtMGD2* and *AtMGD3* (At5g20410 and At2g11810, [36]) were expressed in the SIV PT, 4 h PT, 0.5 h PT and dry pollen transcriptomes (Table S5).

Gene Ontology (GO) term enrichment analysis of the three sectors (S1–S3) also revealed that certain GO term categories are overrepresented in all pollen conditions relative to the whole genome (first 15 GO categories, Figure 2C). The two pollen tube transcriptomes shared 273 genes not found in [dry pollen, 0.5 h PT] (S1, Figure 2B). GO term category overrepresentation analysis also highlighted the overlap between the 4 h PT and SIV PT transcriptomes: genes encoding kinases, antiporters, nucleoside triphosphatases, calcium ion binding proteins, and nucleic acid binding proteins are overrepresented in 4 h PT and SIV PT but not in dry pollen and 0.5 h PT (Figure 2C). Interestingly, the number of genes detected only in SIV PT (1,254) was significantly higher than the number of genes detected only in 4 h PT (75, Figure 2B). These data suggest that growth through the pistil elicits a significant change in the pollen tube transcriptome.

### A Distinct Set of Genes Define Pollen Tube Growth *In Vitro* and in a Pistil

We next explored the overlap in expression between sporophytic tissues (expressed in any of seven sporophytic samples analyzed), SIV PT, and all other pollen conditions (Figure 2D). Notably, SIV PT and sporophytic tissues share a set of 871 genes that are not expressed in the three other pollen samples analyzed. This analysis also identified 2,040 genes that were expressed in pollen but not sporophytic samples. Among these 2,040 genes, 1,097 are shared by all four pollen conditions (Figure 2E). Our analysis also identified a set of 507 pollen tube-enriched genes, including the 100 genes that are common to SIV PT and 4 h PT (Figure 2E). Interestingly, SIV PT has the largest number of unique genes (383) compared to any other pollen condition (Figure 2E and Table S8; referred henceforth as SIV PT-enriched genes), further confirming that the SIV PT transcriptome is distinct from dry pollen or *in vitro* grown pollen tubes despite the overlap it shares with these transcriptomes.

### Genes with Potential Functions in Signal Transduction, Pollen Tube Growth, and Transcription Are Overrepresented Among SIV PT-Enriched Genes

We determined if any GO terms were significantly overrepresented among the 383 SIV PT-enriched genes (Figure 2D, Table S8) compared to pollen-expressed genes (ATGE_73A-C; [28]). Twenty-one GO terms, including those related to signaling, cell extension and transcription, were significantly overrepresented in the SIV PT-enriched genes (P value cut off <0.05, Table 1 and Table S9). The most overrepresented terms in the three GO categories were transmembrane receptor activity (molecular function, n = 4, P = 0.001), defense response (biological process, n = 7, P = 0.003), and intrinsic to membrane (cellular component, n = 4, P = 0.003). There were four genes common to each of these three GO categories and all of them belong to the TIR-NBS-LRR receptor subfamily that is part of a ‘R’ gene superfamily implicated in pathogen recognition [27]. In addition, a set of protein kinases (molecular function, n = 4, P = 0.024) was enriched in SIV PT (Table 1, Table S9). These signaling genes may facilitate pollen tube perception and response to pistil guidance cues. A set of genes annotated as polygalacturonases, sucrose transporters, and antiporters are overrepresented in SIV PT compared to pollen (Table 1, Table S9). These categories have been implicated in pollen tube extension [37]–[42]. Several GO terms related to transcription were also overrepresented in SIV PT-enriched genes (Table 1); they may respond to growth through the pistil and function as key regulators of expression of other genes required for pollen tube growth and guidance (Table 2).

**Table 1:** Overrepresentation of functional categories in SIV PT-enriched genes.

GO term^a^	GO category^a^	GO term counts in		P value^d^	Number of genes present in sperm^e^
		SIV PT-enriched genes^b^	Pollen-expressed genes^c^		
Transmembrane receptor activity	MF	4	7	0.001	0
DNA binding	MF	26	261	0.002	14
Polygalacturonase activity	MF	4	14	0.010	0
Histone acetyltransferase activity	MF	2	2	0.014	0
ATP binding	MF	16	165	0.016	8
Sucrose:hydrogen symporter activity	MF	2	3	0.022	0
Protein kinase activity	MF	4	19	0.024	1
Transcription factor activity	MF	20	237	0.028	9
Nucleoside-triphosphatase activity	MF	3	11	0.029	1
Monovalent cation:proton antiporter activity	MF	3	12	0.035	0
Sodium:hydrogen antiporter activity	MF	3	14	0.048	0
Defense response	BP	7	32	0.003	1
Chromosome segregation	BP	2	2	0.014	2
Sucrose transport	BP	2	2	0.014	0
Regulation of transcription	BP	15	155	0.020	8
Regulation of cell cycle	BP	4	20	0.028	3
DNA repair	BP	4	21	0.032	3
Intrinsic to membrane	CC	4	9	0.003	0
Transcription factor complex	CC	2	2	0.014	2
Ubiquitin ligase complex	CC	4	20	0.028	2
Nucleus	CC	35	479	0.030	19

a GO category classifications: MF, Molecular Function; BP, Biological Process; CC, Cellular Component.

b 349 SIV PT-enriched genes (subset of Table S8; Materials and Methods).

c 6741 pollen-expressed genes (from Table S5).

d Determined by Fisher exact test by comparing SIV PT-enriched and pollen-expressed genes; complete results are provided in Table S9.

e Number of SIV PT-enriched genes scored ‘present’ in sperm [20].

**Table 2:** Significant changes in gene expression profiles among different pollen conditions.

Comparison	Significant changes^a^	UP	Maximum fold UP^b^	DOWN	Maximum fold DOWN^c^
0.5 h PT v dry pollen	23	15	7.2	8	7.9
4 h PT v 0.5 h PT	137	137	45.2	0	0.0
4 h PT v dry pollen	197	186	61.0	11	12.0
SIV PT v dry pollen	1578	1222	382.4	356	28.7
SIV PT v 4 h PT	1135	900	128.2	235	19.0

a Genes that had a B value >3 and were at least three fold different between the two indicated conditions (from the list of genes in Table S5).

UP, the number of genes with significant changes that were greater in the first condition of the comparison relative to the second.

b The highest fold increase in expression for a gene in each comparison.

DOWN, the number of the number of genes with significant changes that were lower in the first condition of the comparison relative to the second.

c The highest fold decrease in expression for a gene in each comparison.

### A Modest Number of Gene Expression Changes Occur During Pollen Hydration and Pollen Tube Growth *In Vitro*

We used a t-test (Materials and Methods) on the RMA-normalized data (Table S5) to define statistically significant changes in gene expression during pollen tube growth (Table 2). To minimize false positives, we established two stringent cut-off values: only those genes that had a B value (false discovery rate) of 3 or higher and a fold change of at least 3 were considered to have undergone a significant change in expression (Table S5).

We analyzed changes that occurred between dry pollen and 0.5 h PT to assess the impact of pollen hydration on gene expression. This analysis defined a very small number of genes that increase (15) or decrease (8) during the hydration process (Table 2, Table S10). To define the changes in transcript levels that occur after hydration and during pollen tube growth *in vitro*, we compared 0.5 h PT and 4 h PT; this time period accounts for nearly all of pollen tube extension observed *in vitro* (Figure 1). One hundred thirty-seven genes had significant increases in expression value in this comparison, while no genes were observed to have a significant decrease (Table 2, Table S11). We also compared dry pollen with 4 h PT and identified 186 genes that increase and 11 genes that decrease during the entire process of hydration and growth *in vitro* (Table 2, Table S12). These results are also consistent with a recent report that identified modest, but significant, changes in the transcriptome of *in vitro*-grown pollen tubes [21].

### A Large Number of Gene Expression Changes Occur When Pollen Tubes Grow Through Pistil Tissues

The number of genes whose expression was significantly different between SIV PT and dry pollen (1,578) or between SIV PT and 4 h PT (1,135) was dramatically greater than any other comparison among the pollen transcriptomes (Table 2, Table S13, Table S14). We compared SIV PT with 4 h PT and identified a large number of genes (900) with significantly higher expression values in SIV PT compared with 4 h PT. There were also a significant number of genes whose expression went down (235) in this comparison (Table 2, Table S13, Table S14). The large number of genes (1,135) that are altered when pollen tubes grow through pistil tissues are candidate factors that underlie the physiological and molecular changes in pollen tubes during a successful fertilization event [22]–[26]. Among the altered genes, we identified a set of genes that can best distinguish SIV PT from dry pollen and 4 h PT (Table S15) using the non-hierarchical k-means clustering method ([43]; also see Materials and Methods). These genes could be used as markers for pollen tubes that have interacted with the pistil.

In SIV PT, the up-regulated genes (compared to both dry pollen and 4 h PT) included the overrepresented molecular function GO categories of transporter, antiporter, symporter activity and calcium ion binding. These functions are known to be critical for pollen tube growth [39]–[42]. Interestingly, a different set of transporter genes is down-regulated in SIV PT compared to 4 h PT; similarly, a separate set of antiporter genes is also down regulated in SIV PT compared to pollen (Figure 2F). These results suggest that transporter and antiporter gene expression is highly dynamic during pollen tube growth in a pistil. There is a significant down regulation of a distinct set of pectinesterases in SIV PT compared to 4 h PT (Figure 2F). Pectinesterases alter the mechanical strength and rigidity of the pollen tube wall during the process of pollen tube elongation [35],[44],[45]; our data suggest the possibility of functional specialization within this large gene family and that, as with transporters, expression of pectinesterases is dynamic in pollen tubes.

### Validation of Pollen Tube Gene Expression Profiling

**Figure 3:**
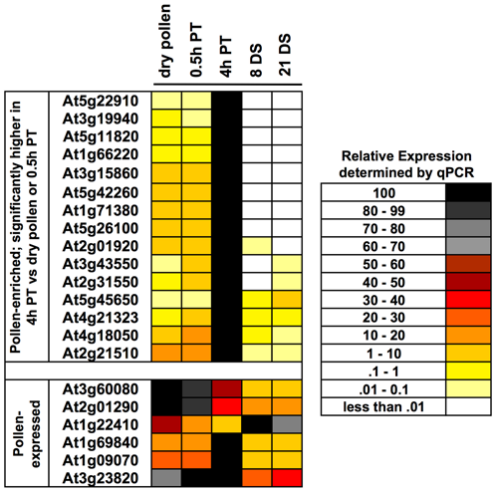
qRT-PCR analysis of 21 pollen tube–enriched and pollen tube–expressed genes. Total RNA from indicated tissues—dry pollen (pollen), 0.5 h PT, 4 h PT, 8- and 21-day-old seedlings (DS)—was analyzed by qRT-PCR. A heat map of the relative expression levels of the indicated genes by qRT-PCR is provided. Expression levels used for the heat map represent an average of gene expression values from four independent qRT-PCR reactions (two technical replicates each for two biological replicates). Relative expression is represented by a color scale that ranges from black (100%) to light yellow (0.01–0.1%). White represents relative expression levels that were below 0.01%. For each gene, the tissue which showed maximum expression was considered 100% (black), and the relative expression in the other four tissues was calculated based on this maximum level.

We used qRT-PCR to verify pollen tube gene expression data obtained from microarray experiments (Materials and Methods). We selected 15 pollen-enriched genes that had high levels of expression in pollen compared to sporophytic tissues and were expressed at significantly higher levels in 4 h PT compared to either dry pollen, 0.5 h PT, or sporophytic tissues (Table S11, Table S12). qRT-PCR corroborated the microarray analysis, showing that all 15 of these genes were pollen-enriched and expressed at significantly higher levels in 4 h PT compared to either dry pollen or 0.5 h PT (Figure S2A, Figure 3, and Figure S3A, first 15 genes). Based on these results, we conclude that our microarray results accurately reflect gene expression patterns during *in vitro* pollen tube growth.

We also tested a set of genes shown by microarray to be expressed at varying levels in pollen, pollen tubes and sporophytic tissues (n = 6) (Table S5). qRT-PCR confirmed the expression of these genes in these cells and tissues (Figure S2A, Figure 3, and Figure S3A, bottom six genes). The relative expression of At3g60080, At2g01290, and At1g22410 were identical in qRT-PCR and microarray experiments (Figure 3, Figure S3A). Expression of the remaining three genes (At1g69840, At1g09070, At3g23820) was confirmed in pollen and sporophytic tissues by qRT-PCR; however, there were discrepancies in the relative expression of these genes when qRT-PCR data were compared with microarray experiments (Figure 3, Figure S3A). These differences could be attributed to the variability in plant growth and RNA preparation from 8-day- and 21-day- old seedlings between different laboratories (our data and that of [28]).

We next determined whether the gene expression differences between SIV PT and 4 h PT in microarray experiments (Table S14) could be detected by qRT-PCR by testing 10 genes with expression values that were significantly higher, and six genes that were significantly lower, in SIV PT compared to 4 h PT (Figure S2B, Table 3, Table S14). All 10 genes expressed at higher levels in SIV PT compared to 4 h PT in microarray experiments were also higher in SIV PT by qRT-PCR experiments (Table 3). The reduction in gene expression detected by microarray for six genes was also confirmed by qRT-PCR (Table 2). Based on these results, we conclude that a high degree of confidence can be placed on the changes in gene expression identified by the microarray experiments reported in this study.

**Table 3:** qRT-PCR validation of significant gene expression differences between SIV PT and 4 h PT.

**Up-regulated genes in SIV PT**					
**Gene ID**	**FC microarray** a **(SIV PT/4 h PT)**		**qRT-PCR (SIV PT/4 h PT)**		
			**FC** b		**Standard deviation**
At1g09080	128.18		150.87		73.78
At1g63530	27.36		22.25		18.75
At2g17000	21.47		8.23		1.66
At1g72760	20.78		13.96		6.59
At1g18830	11.33		10.88		1.33
At4g35710	11.10		17.53		5.22
At1g66570	10.89		165.25		79.87
At2g04230	9.38		23.05		12.89
At1g80870	8.54		8.83		2.61
At3g18000	4.92		1.25		0.54
**Down-regulated genes in SIV PT**					
**Gene ID**	**FC microarray** a **(4 h PT/SIV PT)**	**qRT-PCR (4 h PT/SIV PT)**			
		**FC** b		**Standard deviation** c	
At5g28470	19.03	>10000^d^		>10000^d^	
At5g27870	12.64	500.75		169.85	
At3g06830	8.39	79.34		23.06	
At1g73630	8.09	21.01		9.94	
At2g28080	5.55	1015.77		539.74	
At3g01820	5.46	41.05		18.26	

a Average fold change in expression of indicated genes by microarray experiments (from Table S14).

b Average fold change in gene expression between SIV PT and 4 h PT conditions calculated from four independent qRT-PCR reactions (two technical replicates each of two biological replicates).

c Standard deviation of fold change in expression.

d This gene was not expressed in SIV PT in all four replicates of qRT-PCR experiments, and, as described in Materials and Methods, a C_T_ value of 45 was assigned resulting in extremely high fold change and standard deviation values.

### Confirmation of Pistil-Dependent Changes in Pollen Tube Gene Expression *In Vivo*

**Figure 4:**
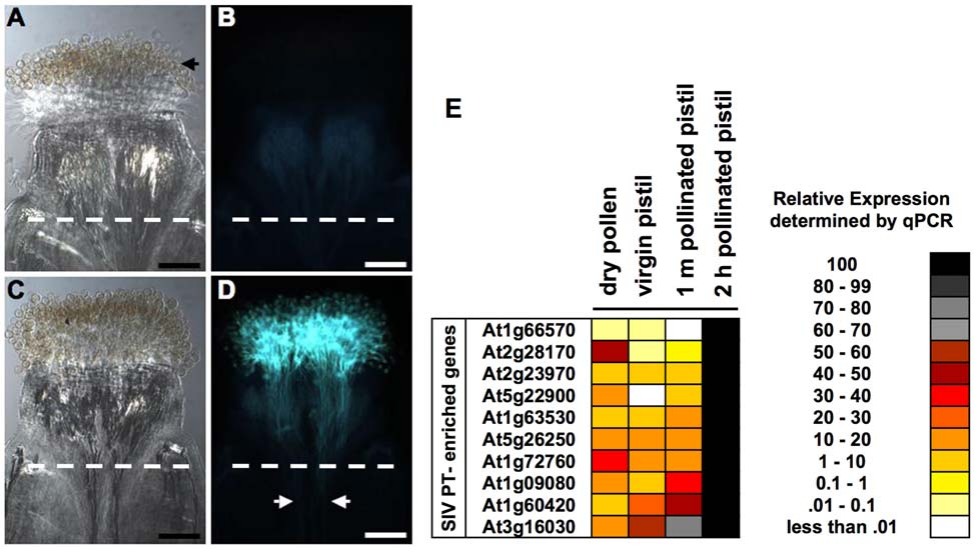
*In vivo* confirmation of pistil-dependent changes in pollen tube gene expression. (A–D) Aniline blue staining of pollen tube growth in pistils. (A and C) are bright field images; (B and D) are epifluorescent images. (A and B) Pollinated pistils stained with aniline blue soon after pollen deposition. Tubes have not emerged from the grains but adhesion to stigmatic cells can be seen (black arrow). (C and D) Pollinated pistils stained with aniline blue 2 hours after pollen deposition; the pollen tube front in the style is indicated by white arrows. (E) A heat map of the relative expression levels (as described in Figure 3) measured by qRT-PCR of the indicated genes in dry pollen, emasculated *ms1* pistils, pollinated pistils 1 minute (1 m pollinated pistil) or 2 hours (2 h pollinated pistil) after pollen deposition. Pistils were cut at the junction of the style and ovary (white dotted line); the portion with the stigma and style (B), or stigma, style, and pollen tubes (D), were used in qRT-PCR experiments (E). Scale bars, 100 µm.

To determine whether pollen tube growth in an intact pistil elicits similar changes in gene expression as those observed in microarray analysis of SIV PT, we used qRT-PCR, to monitor gene expression changes between dry pollen, unpollinated pistils, pistils pollinated for one minute, and pistils pollinated for two hours (an *in planta* tissue type that most closely resembles SIV PT). We evaluated expression of a set of genes that were found to be induced in SIV PT compared to dry pollen by microarray experiments (Table S13). Pollen tubes comprise only a small fraction of pollinated pistil tissue; therefore, we undertook two strategies to allow detection of induction of gene expression in *in vivo*-grown pollen tubes. First, among the genes induced in SIV PT, we chose genes with relatively low expression in the stigma and ovary [29] (Table S5), as high expression in these tissues would preclude detection of induction in pollen tubes. Second, we isolated RNA from the stigma and style portion of the pollinated pistil because pollen tubes are concentrated here during the first two hours of growth (Figure 4A). qRT-PCR shows that the mRNA abundance of all 10 genes was higher in pistils that had been pollinated for two hours compared to pistils that had been pollinated for one minute, unpollinated pistils, or dry pollen (Figure 4B). These results suggest that increases in mRNA abundance detected in SIV PT also occur during pollen tube growth in a pistil.

### Reverse Genetic Analysis of Genes Induced During Pollen Tube Growth

We used reverse genetic analysis of 33 genes that were significantly higher in SIV PT versus 4 h PT (n = 10, range = 5 to 22 fold change, Table S14) or 4 h PT versus dry pollen (n = 23, range = 3 to 41 fold change, Table S10) to determine whether candidate genes identified by microarray analysis were critical for pollen tube growth and guidance. T-DNA insertion mutants from the Syngenta *Arabidopsis* Insertion Lines (SAIL, [46]; Table S16) were analyzed using a series of sensitive pollen function assays.

A subset of the SAIL collection [46] was generated in the *quartet (qrt)* mutant background with a T-DNA carrying a β-glucuronidase (GUS) reporter gene expressed from the pollen-specific LAT52 promoter [46]–[48] and a Basta (herbicide) resistance gene. The *qrt* mutation causes the four products of male meiosis to be released as a tetrad of pollen grains but does not interfere with pollen tube growth [48]. These unique features of the SAIL collection offer significant advantages for analysis of pollen mutant phenotypes over other mutant collections. First, mutant pollen grains can be easily identified within tetrads produced by a heterozygous mutant plant (2 GUS+ mutant: 2 GUS- wild type). Second, GUS expression in mutant pollen tubes allows direct comparison between mutant and wild-type pollen tube growth *in vitro* or in a pistil. These attributes have been exploited previously in forward genetic analysis of pollen tube growth and guidance [5],[49].

The pollen function assays we employed require cosegregation between Basta resistant (Basta) in seedlings, GUS expression in pollen, and a single locus T-DNA insertion in the gene of interest. We identified 50 SAIL lines with potential insertions in 33 genes chosen for analysis (Table S16). Using PCR, we verified T-DNA insertion sites in Basta progeny from 39 of the 50 lines (Table S16). Twenty-seven of these 39 insertion lines showed 2 GUS+ (mutant): 2 GUS- (wild type) segregation in pollen tetrads and were heterozygous for the insertion by a PCR assay (Table S16 and Materials and Methods), indicating cosegregation between the gene of interest and the T-DNA insertion. We discarded the other 12 lines because GUS expression in pollen tetrads was consistent with multiple T-DNA insertion sites (Table S16). To further confirm that the remaining 27 lines had a single-locus insertion of the T-DNA, we analyzed the segregation of Basta among the progeny of a self-fertilized heterozygous plant (2∶2 GUS+: GUS- tetrads, heterozygous by PCR assay). Plants heterozygous for a single insertion site are expected to generate 75% Basta progeny (3∶1 segregation of dominant marker). However, if the insertion disrupts a gene required for male/female gametophyte function, or seed development, the fraction of Basta progeny will be significantly reduced [50]. We found that the percentage of Basta progeny was ∼75% or significantly lower in all 27 lines, confirming that they had a single T-DNA insertion site and indicating that several (16/27) may disrupt the male and/or female gametophyte or seed development (Table 4).

**Table 4:** Transmission of insertion in self-fertilization and male crosses.

Insertion	FC^a^	% Basta (self)	n (self)	% Basta (♂ cross)	n (♂ cross)
Control heterozygote	-	75.12	414	51.40	358
**Genes significantly up regulated in 4 h PT vs dry pollen**					
At2g31550-1	41.17	68.92*	1036	50.53	283
At2g25630-1	18.13	62.63*	643	55.19	270
At5g22910-1	17.81	73.41	361	49.41	506
At5g23530-1	15.92	72.12	624	48.40	219
At4g18050-1	13.87	41.19*	437	57.64	203
At4g18050-2	13.87	73.67	338	52.53	198
At4g18050-3	13.87	76.50	200	49.13	230
At4g18050-4	13.87	69.89	269	48.43	159
At4g21323-1	13.77	63.26*	596	53.96	194
At5g12030-1	11.43	80.75	265	50.61	326
At1g74450-1	10.55	61.07*	763	58.85	209
At1g60420-1	10.16	69.06*	934	37.58*	753
At4g08670-1	7.05	42.80*	236	53.96	278
At2g05160-1	5.15	66.39*	598	42.54	268
At2g05160-2	5.15	61.74*	345	51.34	187
At5g55020-1	3.77	59.24*	920	54.78	115
At5g59720-1	3.63	70.98	224	46.13	388
At2g01920-1	3.38	79.45	253	45.87	508
At1g79360-1	3.06	75.57	221	45.92	331
**Genes significantly up regulated in SIV PT vs 4 h PT**					
At2g23970-1	22.10	53.50*	286	40.78	206
At1g55910-1	16.78	62.01*	437	50.08	645
At1g72150-1	16.75	70.90	354	50.39	381
At5g28540-1	11.93	66.67*	372	49.15	413
At5g66890-1	11.02	73.71	350	55.71	219
At2g34920-1	8.45	64.86*	370	52.40	250
At5g67250-1	7.67	68.89*	389	48.06	283
At3g18000-1	4.93	28.94*	622	36.21*	649

a FC, fold change in gene expression levels in microarray experiments (from Table S12 and Table S14).

% Basta, the percentage of Basta resistant F1 progeny from either self-fertilization (self) or *ms1* female X heterozygous insertion male (♂ cross).

n, number of progeny plants scored on Basta plates.

*: Significantly different from expected (75% in self or 50% in male crosses), (χ^2^, *P* <0.01).

### At1g60420-1 and At3g18000-1 Reduce Transmission Through Pollen

Mutations that completely disrupt pollen function are not transmitted to progeny through pollen, while milder defects reduce, but do not eliminate transmission [49]. To focus on transmission of the T-DNA through pollen, we pollinated *male sterile 1* (*ms1*) pistils with heterozygous pollen from 27 single-locus T-DNA insertion lines and determined the percentage of Basta plants in the progeny (Table 4). Any significant deviation from 50% in this assay indicates that mutant pollen is less likely to fertilize ovules than wild-type pollen. We found that progeny from two of the insertion lines, one in a 4 h PT-induced gene (At1g60420) and another in a SIV PT-induced gene (At3g18000) yielded significantly fewer than the expected 50% Basta plants (Table 4) indicating that these genes are critical for pollen function in the pistil.

At1g60420 encodes an uncharacterized protein with thioredoxin and C1-like domains. C1 domains have been shown to bind diacylglycerol and phorbol esters and are implicated in lipid signaling in mammals [51]. At3g18000 *(XIPOTL)* encodes one of three *Arabidopsis* S-adenosyl-L-methionine: phosphoethanolamine N-methyltransferase (PEAMT) required for synthesis of phosphatidylcholine, a major membrane lipid and the precursor of phosphatidic acid, an important lipid signaling molecule [52]. Since the proteins encoded by these two genes may be involved in generation of lipid signaling molecules, the *in vivo* transmission defects of insertions in these genes point to a potential role for lipid signaling in pollen tube growth through the pistil.

### At1g60420-1 and At3g18000-1 Cause Defective Pollen Tube Growth in the Pistil

**Figure 5:**
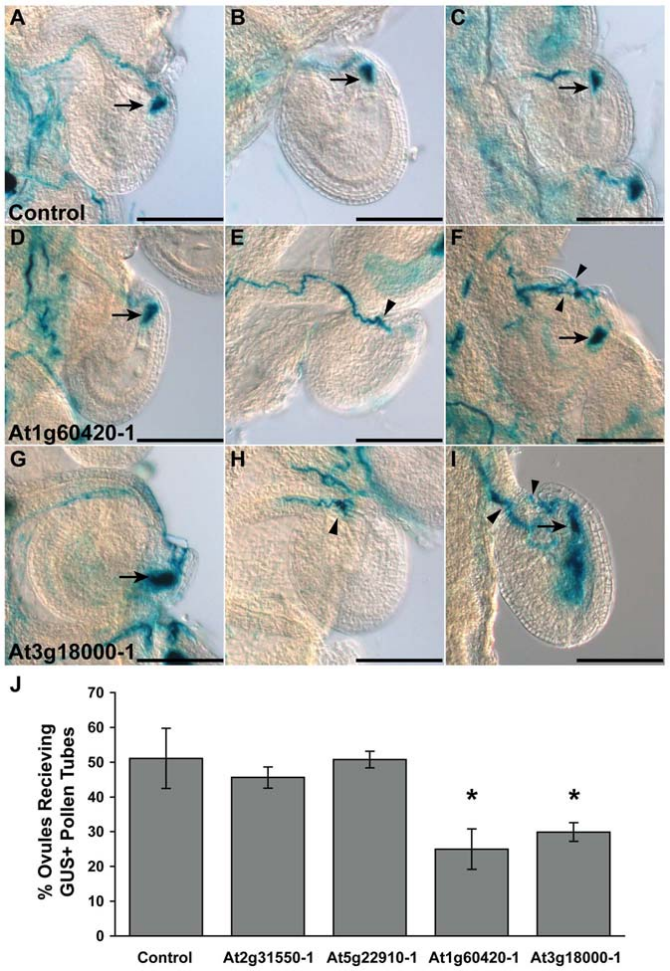
At1g60420-1 and At3g18000-1 cause defective pollen tube growth in the pistil. (A–I) *ms1* pistils were hand-pollinated with pollen heterozygous for At1g60420-1, At3g18000-1, or a control insertion (does not affect pollen function), and were stained for GUS activity 24 hours after pollination. GUS is released into synergids from pollen tubes that successfully enter the micropyle and burst (arrows). (A–C) Ovules that have received GUS+ control pollen tubes. (D,F,G,I) Ovules that have received GUS activity from pollen tubes carrying the indicated insertion. (E,H) Some ovules attracted pollen tubes that failed to target the micropyle and burst. (F,I) Some ovules received GUS activity and have multiple additional tubes targeting the micropyle. Scale bars = 50 µm. (J) Quantitative analysis of ovule targeting. The number of ovules that received GUS activity following pollen tube burst was quantified for the control and indicated insertion lines and is plotted as a percentage (±s.d.) of the total number of ovules. *P value>0.01 (χ^2^, expected = 50%).

To analyze the growth behavior of At1g60420-1 and At3g18000-1 pollen tubes *in vivo* and determine the specific stage of pollen tube growth disrupted by these insertions, we pollinated *ms1* pistils with heterozygous pollen and stained for GUS activity 24 hours later [5],[49]. When *ms1* pistils were pollinated with heterozygous control pollen, GUS+ pollen tubes germinated, penetrated the stigmatic papillae, grew through the style, entered the ovary through the transmitting tract, and migrated toward an ovule. After entering the micropyle, GUS+ pollen tubes burst, releasing an aggregate of GUS activity in the micropylar end of the ovule serving as a convenient marker for successful ovule targeting by a pollen tube (Figure 5A–5C). In this assay, ∼50% of ovules were targeted by GUS+ pollen tubes from the heterozygous control line (Figure 5J; [5]). When *ms1* pistils were pollinated with heterozygous At2g31550-1 or At5g22910-1 pollen (insertions that did not affect mutant allele transmission through pollen, Table 4) the germination and growth of the GUS+ tubes in stigma, style and transmitting tract was normal (data not shown) and nearly 50% of the ovules were targeted by GUS+ pollen tubes (Figure 5J). However, when *ms1* pistils were pollinated with heterozygous At1g60420-1 or At3g18000-1 pollen, GUS+ pollen tubes were only half as efficient in targeting ovules as the GUS- tubes (Figure 5D, 5G, 5J). These results are consistent with the reduction in mutant allele transmission in At1g60420-1 and At3g18000-1 insertion lines (Table 4).

In addition to a significant reduction in the ability to target ovules, At1g60420-1 and At3g18000-1 GUS+ pollen tubes exhibited an increased frequency of abnormal pollen tube behaviors. Unlike in control crosses (*ms1* ovules with GUS+ pollen tubes from control heterozygotes, 0%, n = 182), a noticeable fraction of *ms1* ovules had At1g60420-1 or At3g18000-1 GUS+ pollen tubes that approached, but did not enter, the ovule micropyle (Figure 5F, 5I; At1g60420-1, 8.33%, n = 204; At3g18000-1, 5.49%, n = 164). For a small number of ovules, pollen tubes grew towards the chalazal end, instead of the micropylar end, of the ovule (not shown; At1g60420-1, 1.22%, n = 204; At3g18000-1, 1.96%, n = 164; control, 0%, n = 182). Finally, ovules that attracted multiple GUS+ At1g60420-1 or At3g18000-1 pollen tubes were observed (Figure 5E, 5H; At1g60420-1, 1.83%, n = 204; At3g18000-1, 3.43%, n = 164; control, 0%, n = 182).

### Five Insertions Disrupt Pollen Tube Growth *In Vitro*

We wanted to determine whether At1g60420-1 or At3g18000-1, insertions that disrupt pollen tube growth in the pistil, had inherent defects in pollen tube extension. We also wanted to examine if other single-locus insertion lines had subtle defects in the ability of pollen grains to form and extend a polar tube that may have been masked by growth in the pistil. *In vitro* pollen germination and tube growth provides a sensitive and direct assay for pollen function that is independent of pistil tissue. We assayed *in vitro* pollen tube germination and growth for the 27 single-locus insertion lines. We used heterozygous pollen so that we could analyze mutant pollen (GUS+, blue) alongside wild-type pollen (GUS-, white) after staining for GUS activity. This side-by-side comparison between mutant and wild type is critical because it provides an internal control for the inter-experiment variability of pollen tube growth *in vitro* [53]. GUS staining in pollen tubes was dark enough to clearly distinguish mutant from wild type in 12 lines (Table 5, Figure S4).

**Table 5:** Reverse genetic analysis identifies insertional mutants that disrupt pollen tube growth *in vitro*.

Insertion	GUS	Germ^a^ (%)	n	P value	Length^a^ (µm)	n	P value
Control	+	23.14	1432	NS	202.07	282	NS
	−	22.80	1432		197.13	381	
**4 h PT**							
At2g31550-1	+	5.25	1110	0.0107	65.23	152	<0.001*
	−	32.29	1110		216.66	331	
At5g22910-1	+	46.07	680	NS	228.16	228	0.0393
	−	53.92	680		257.22	213	
At5g23530-1	+	26.43	914	0.0124	119.28	145	<0.001*
	−	58.48	914		298.42	167	
At4g18050-4	+	58.26	526	0.0052	217.04	212	NS
	−	62.17	526		233.11	222	
At4g21323-1	+	23.01	580	0.0352	181.79	177	NS
	−	20.42	580		194.19	180	
At1g60420-1	+	17.40	686	NS	206.98	133	NS
	−	20.23	686		188.57	177	
At4g08670-1	+	10.25	568	0.0300	110.04	140	<0.001*
	−	30.66	568		220.72	261	
At5g55020-1	+	5.06	718	<0.001*	62.46	100	<0.001*
	−	36.13	718		174.63	273	
At5g59720-1	+	44.44	766	0.0772	270.83	215	0.0169
	−	54.96	766		316.97	240	
**SIV PT**							
At1g72150-1	+	58.86	610	0.0175	176.04	302	NS
	−	63.00	610		175.09	322	
At5g67250-1	+	9.95	1144	0.0271	159.85	227	<0.001*
	−	29.45	1144		215.60	345	
At3g18000-1	+	26.41	720	NS	199.15	153	0.007
	−	27.54	720		234.49	152	

GUS, β-glucuronidase staining present (+) or absent (−) in pollen grains or tubes.

% germ, percentage of pollen grains that germinated.

a Least squares means are reported for % germination (germ) and pollen tube length (length).

n, number of pollen grains or pollen tubes analyzed.

P value, statistical analysis of separation between least squares means.

*: P values <0.001 were considered to be statistically significant.

NS, P value >0.05.

4 h PT, genes significantly higher in 4 h PT compared to dry pollen in microarray experiments.

SIV PT, genes significantly higher in SIV compared to 4 h PT in microarray experiments.

We analyzed at least three replicates of all *in vitro* pollen germination and tube length experiments using statistical methods that account for variation between and within experiments and set a stringent criterion for statistical significance at P<0.001. *In vitro* pollen germination rates and tube lengths were similar for GUS+ and GUS- pollen from heterozygous control plants (Table 5, Figure S4). Although At1g60420-1 and At3g18000-1 were transmitted through the pollen with significantly reduced frequencies (Table 4) and were less likely to target ovules (Figure 5), these insertions did not affect tube growth *in vitro* (Table 5, Figure S4). These results indicate that the *in vivo* transmission defect in these insertion lines cannot be due to an inherent defect in pollen tube extension and is likely caused by loss of functions specifically required to navigate the pistil environment.

The lengths of GUS+ pollen tubes were significantly shorter (P<0.001) than GUS- pollen tubes for insertions in At2g31550 (GDSL-motif lipase/hydrolase family protein), At5g23530 (carboxyesterase 18), At4g08670 (similar to lipid transfer proteins), At5g67250 (an SCF-type F-box and leucine rich repeat-containing E3 ubiquitin ligase), and At5g55020 (*MYB120*, Table 5, Figure S4). Of the 12 lines we analyzed, only the insertion in *MYB120* (At5g55020-1) also caused a significant defect (P<0.001) in pollen tube germination (Table 5, Figure S4). These results indicate that insertions in five genes resulted in pollen tube growth defects that were only detectable *in vitro*.

## Discussion

### The Pollen Tube Transcriptome Changes Significantly Following Growth Through Pistil Tissue

We characterized the global gene expression profiles of *in vitro-* and semi *in vivo-*grown *Arabidopsis* pollen tubes and present the first molecular and genetic analysis of a set of genes expressed by the pollen tube as it grows through pistil tissue. One approach to identify pollen tube genes that respond to the pistil would be to isolate intact, in vivo-grown pollen tubes. This is possible in species like lily that have a hollow style [54],[55]; however, genomic resources are not currently available for these plants. In Arabidopsis, pollen tubes grow deep within a solid style, making it extremely difficult to obtain sufficient quantities of pure *in vivo*-grown pollen tubes for microarray analysis. We overcame this challenge by collecting a large number of pollen tubes that had grown through pistil tissue using the semi-*in vivo* method [26]. This procedure offered several advantages over alternative methods. First, harvested pollen tubes were directly used for RNA isolation without any further manipulations (such as cell sorting or protoplast preparation). Second, it allowed the wild-type pollen tube transcriptome to be assessed directly and eliminated the need for using mutant or transgenic marker lines that could have inappropriately altered the dynamics of wild-type pollen tube gene expression. Third, our method enriches for the actively extending pollen tube tip, which includes the vegetative nucleus, two sperm cells, and majority of the pollen tube cytoplasm. Finally, because SIV PT is comprised solely of pollen tubes, we were able to detect even those genes that i) are expressed at low levels in pollen tubes, ii) exhibited pollen tube-specific expression, and iii) undergo only modest changes in expression during pollen tube growth through the pistil.

Characterization of the SIV PT transcriptome provides the first global view of pistil-dependent gene expression changes in pollen tubes. The SIV PT transcriptome is about 10% (∼700 genes) larger than the pollen or *in vitro*-grown pollen tube transcriptome (Figure 2). The pollen tube gene expression profile undergoes a dramatic change upon interaction with the pistil; expression levels of nearly 1,500 and 1,100 genes are significantly altered in SIV PT compared to dry pollen and 4 h PT, respectively (Table 2). Finally, a distinct set of transcripts accumulate preferentially in SIV PT relative to pollen, 4 h PT and sporophytic tissues (Table S13); defining these genes offers an opportunity to further investigate the molecular basis of the pollen tube response to the pistil environment. Additional analysis will be necessary to examine changes in the pollen tube transcriptome elicited by other pistil tissues such as the transmitting tract and ovules.

### Overrepresentation of Genes Involved in Signaling, Pollen Tube Growth, and Transcription in SIV PT-Enriched Genes

All of the genes that were annotated as transmembrane receptors and overrepresented in the SIV PT-enriched gene list were TIR-NBS-LRR-type receptor proteins (Table 1), a subgroup of the resistance (R) gene family that mediate molecular recognition of pathogen-derived effector proteins [27],[56]. The precise biological functions of many members of this large gene family, including the four in the SIV PT-enriched gene list, have not been determined. However, it is clear that some TIR-NBS-LRR-type genes have functions unrelated to plant defense. For example, an *Arabidopsis* TIR-NBS-LRR-type receptor mutant (At5g17880) has a constitutive shade-avoidance response [57]. TIR-NBS-LRR receptors are highly variable and show signatures of rapid evolution [27], features common in reproductive proteins that contribute to species-specific interactions between mating partners [58],[59]. Intriguingly, another family of variable proteins, initially identified as defensins, were recently shown to function as pollen tube attractants in Torenia [10]. It will be interesting to explore the function of TIR-NBS-LRR receptors in pollen tube growth and guidance using the genetic approaches described here.

Our microarray analysis identified a large number of mRNAs that increase in abundance as pollen tubes grow *in vitro* or through the pistil (Figure 2B and 2C, Table 2), adding support to the view that pollen tubes transcribe mRNA during pollen tube growth [21]. Several categories of genes involved in transcription were enriched in SIV PT compared to pollen (Table 1). A variety of transcription factors including *MYB65* (see below), other MYB-family proteins (At5g38620, At2g13960, At2g20400), MADS box-containing proteins (At5g38620, *PHERES 2/AGL38, AGL73*), and homeobox-containing proteins (At3g19510, At2g32370) were among the overrepresented genes, suggesting the existence of a network of gene regulatory mechanisms to mediate pollen tube growth in the pistil. Genetic analysis of pollen tube-expressed transcription factors (see below) offers the potential to identify key regulators of pollen tube gene expression.

The SIV PT sample includes the two sperm cells; so, some SIV PT-expressed genes may be transcribed in the sperm nucleus. The transcriptome of sperm cells purified from pollen grains, comprising 5,829 *Arabidopsis* genes, was recently characterized [20]. We examined the overlap between SIV PT-enriched genes and genes called ‘present’ in sperm and found that 161 of the 383 SIV PT-enriched genes (43%) are expressed in sperm (Table 1, Table S7). Genes overrepresented in SIV PT, but not detected in sperm, include those potentially important for signaling (transmembrane receptor activity, Table 1), transcription (histone acetyltransferase activity, Table 1) and pollen tube growth (polygalacturonase, sucrose transport and antiporter activity, Table 1). Genes proposed to be involved in DNA repair, chromosome segregation, and cell cycle regulation (Table 1) were overrepresented in SIV PT-enriched genes; all of these genes are present in sperm (Table 1, Table S8) [20]. The pollen tube nuclear DNA (vegetative nucleus) does not replicate during pollen tube growth; however, sperm complete a round of DNA synthesis during pollen tube growth in the pistil [60]. This group of genes, identified in the SIV PT transcriptome, is therefore likely expressed in sperm as the pollen tube is growing through the pistil and function in sperm DNA synthesis.

### Microarray-Directed Reverse Genetic Analysis of Pollen Tube Growth

One of the goals of this study was to assess the extent to which microarray analysis identifies genes that are critical for pollen function. We identified single-insertion-locus T-DNA lines and employed four highly sensitive assays to determine loss-of-function phenotypes in pollen. Insertions in two genes (At3g18000, At1g60420) affected mutant allele transmission through pollen and disrupted pollen tube growth and guidance *in vivo*. Insertions in five additional genes (At2g31550, At4g08670, At5g23530, At5g55020, At5g67250) caused pollen tube growth defects *in vitro*. A previous forward genetic screen yielded ∼30 mutants that disrupt pollen function from a population of ∼10,000 T-DNA insertion lines (0.3%, [49]). In this study, by starting with a population of 50 T-DNA insertions in genes induced during pollen tube growth, we identified seven mutations that disrupt pollen tube growth *in vitro* or in the pistil (14%); this amounts to a ∼45 fold enrichment in identification of functionally significant genes over the forward genetic screen.

We ascribed loss-of-function mutant phenotypes to seven *Arabidopsis* genes not previously implicated in pollen tube growth (Figure S3B). Five of these genes (At2g31550, At4g08670, At5g23530, At5g55020, At1g60420) were not characterized genetically before this study. In these mutants, we confirmed that the T-DNA disrupted the gene of interest using gene-specific PCR and showed that this PCR product cosegregated with two reporter genes (Basta and GUS expression) carried on the T-DNA. All pollen assays directly compare the function of pollen with the T-DNA insert (GUS+ and carrying Basta gene) with wild-type pollen (GUS- and not carrying Basta gene) as they were performed in pollen tetrads from heterozygous plants. The mutant phenotypes we identified are linked to the T-DNA insertion. Therefore, we can rule out the possibility that unlinked mutations, not tagged by the T-DNA in the gene of interest, are responsible for the observed phenotypes. Our data suggest that loss-of-function of the indicated genes caused the pollen phenotypes recorded here.

In this study, we systematically addressed whether mutations that affect pollen tube growth *in vitro* also disrupt pollen tube growth *in vivo*. It is reasonable to predict that a mutation affecting a pollen tube structural component or a factor required for tip growth would disrupt growth in either the pistil or in a defined growth medium [34],[35],[61]. However, a mutation that specifically disrupts the ability of the pollen tube to re-orient growth in response to pollen tube guidance cues would not be expected to cause a defect in the ability of pollen tubes to extend *in vitro*. We found two insertions (At3g18000-1 [*XIPOTL*]; At1g60420-1 [thioredoxin and C1-domain containing]) that caused significant reductions in ovule targeting (Table 4, Figure 5), but did not affect pollen tube growth *in vitro* (Table 5, Figure S4). A third type of mutation would cause mutant phenotypes *in vitro*, but would not result in defective growth in the pistil environment. These mutations may define genes that play a role in the growth process, but whose mutant phenotypes in the pistil are masked by factors in the pistil environment that enhance growth [24]. For these mutations, *in vitro* pollen tube growth may be viewed as a sensitized environment capable of revealing subtle mutant phenotypes. We identified five insertions that disrupted pollen tube growth *in vitro* that did not obviously affect the ability of pollen to sire progeny *in vivo*. For example, At5g55020-1 (*MYB120*, discussed below) significantly reduced pollen germination and tube length *in vitro* (Table 5, Figure S4), but did not affect transmission of the mutant allele through pollen (Table 4). Our microarray data show that the transcriptome of pollen tubes grown *in vitro* is dramatically different from that of pollen tubes grown through pistil tissue. Our genetic experiments also confirm this difference by showing that the consequences of loss-of-function in a pollen tube gene are different in these distinct environments and that the combination of assays probing growth *in vitro* and *in vivo* is essential to comprehensively understand pollen tube growth.

An insertion in *MYB120* (At5g55020-1) caused defective pollen germination and tube growth *in vitro* (Table 5, Figure S4). Phylogentic analysis of 125 MYB-related transcription factors place *MYB120* in subgroup 18, which comprises seven closely related genes [62],[63]. Analysis of 125 *Arabidopsis* MYBs in our data set showed that three of the four most abundant MYBs in the SIV PT are from subgroup 18 (including *MYB120*). Furthermore, four members of subgroup 18 are expressed at much higher levels in pollen than in other tissues we analyzed and three members (including *MYB120* and *MYB65*) of the subgroup have their peak expression in SIV PT (Figure S5). *MYB65* is a SIV PT-enriched gene (Table S8) and was identified among the transcription factors overrepresented in SIV PT-enriched genes compared to pollen-expressed genes (Table S9). Perhaps functional redundancy within this MYB subgroup explains why an insertion in *MYB120* (At5g55020-1) affected pollen tube growth *in vitro* did not cause a defect in the pistil (Table 4). Analyzing single and multiple mutations in members of this subgroup, using the assays described here, can be used to test this hypothesis and to determine whether this group of transcription factors is an important regulator of gene expression in actively extending pollen tubes.

We uncovered a role in pollen tube growth for two genes (At5g67250, At3g18000) already shown to be critical for sporophytic growth and development. Our microarray analysis shows that both of these genes have broad expression patterns in sporophytic tissues and are significantly higher in SIV PT than 4 h PT (Figure S3B). These expression patterns underscore an important aspect of the SIV PT transcriptome; 871 genes (Figure 2D) are shared between SIV PT and the sporophytic tissues we analyzed that are not expressed in pollen or pollen tubes grown *in vitro*. At5g67250 and At3g18000 illustrate how functional analysis using pollen can provide new insights into the function of this part of the *Arabidopsis* genome.

Previous RNAi analysis of At5g67250 (an SCF-type F-box and leucine rich repeat-containing E3 ubiquitin ligase, *VFB-4*) showed that reduction of expression was associated with defects in lateral root formation and rosette leaf expansion [64]. SCF-type E3 ubiquitin ligases determine substrate specificity for ubiquitination and proteolysis, thereby regulating an array of biological processes including cell cycle progression [65],[66] and auxin signaling [67],[68]. Here we have shown that *VFB-4* is required for pollen tube growth *in vitro* (Table 5, Figure S4), suggesting that regulated proteolysis is important for pollen tube extension. A limitation of microarray analysis is that it only documents changes in mRNA abundance and does not identify genes whose mRNA levels remain unaltered, but encode proteins that undergo post-translational modification in response to growth in the pistil. Post-translational regulation of protein function is likely an important mediator of pollen-pistil interactions. For example, LePRK2, a pollen-specific receptor kinase required for pollen tube growth in tomato [69] has been shown to be dephosphorylated by a stigma extract [70]. Methods developed for large-scale SIV PT isolation (this study) and pollen proteomic analysis [71]–[73], could be combined to identify the set of pollen tube proteins that are modified in response to growth in the pistil.

At3g18000 (*XIPOTL*) was identified in a genetic screen for root architecture defects [52], and encodes a PEAMT required for production of phosphatidylcholine (see Results). At3g18000-1 disrupted ovule targeting in the pistil (Table 4, Figure 5), suggesting that *XIPOTL* may be required for navigating the pistil environment and that lipid signaling and/or a particular plasma membrane composition is required for pollen tube growth and guidance.

## Materials and Methods

### Plant Materials and Growth Conditions

*Arabidopsis* plants were grown in chambers at 21°C under illumination (100 µmol m^−2^ s^−1^ with a 16-hour photoperiod). Wild-type pollen and pollen tubes (Col-0 accession) were used for microarray experiments. The SAIL lines (Col-0 accession) and *male sterile 1* mutant, *ms1* (CS75, Landsberg ecotype) were obtained from the *Arabidopsis* Biological Resource Center (Columbus, OH). *ms1* does not produce pollen, but has a normal pistil; this mutant therefore yields pistils that do not require emasculation.

### Collection of Dry Pollen and *In Vitro–*Grown Pollen Tubes

Dry pollen grains were collected by the vacuum method [74] into microfuge tubes containing 250 µl liquid pollen growth medium [75] and incubated for 0.5 or 4 hours in a 24°C growth chamber. Pollen tubes were centrifuged at 4,000 rpm for 5 minutes, the supernatant was removed and the microfuge tubes were frozen in liquid nitrogen and stored in −80°C until RNA isolation. Aliquots of pollen tube suspensions were observed under an Axiovert 100 microscope (Carl Zeiss, Oberkochen, Germany) to determine % pollen germination and pollen tube length using Metamorph software version 7.1.4.0 (Molecular Devices Inc., Downingtown, PA). Pollen grains with emerging tubes equal to or longer than their diameters were considered germinated. After 4 hours of growth, 58.3±6.0% of the pollen grains germinated and formed tubes (average length of 383.8±32.1 µm; Figure 1C). Under our growth conditions, the remainder of the grains in the 4 hour sample did not germinate and the germination rates did not increase even with longer incubation times (Figure 1C).

### Collection of Semi *In Vivo*–Grown Pollen Tubes (SIV PT)

Pollen tubes grown through the stigma and style were collected by the semi *in vivo* procedure essentially as described [26]. Pollinated *ms1* pistils were placed vertically on solid pollen growth medium for one hour (establish growth into the pistil) before they were laid horizontally; tubes emerged from cut pistils after three hours of growth and were harvested as bundles after three hours of growth on the media surface. Bundles were excised at the point of emergence from the cut pistil and collected into liquid nitrogen-frozen microfuge tubes. Eight hundred pollen tube bundles (obtained from 800 cut pistil explants) were used for each of the three replicate RNA isolations. Pollen tube bundles were confirmed to be free of pistil tissue contamination by microscopy.

### Collection of Other Tissues and Pollinated Pistils

For 8-day-old seedling samples, both shoots and roots of seedlings grown on 0.5X Murashige and Skoog (MS) media [MS salts (Carolina Biological Supply Company, Burlington, North Carolina), 10% sucrose, pH 5.7, 7% Bacto Agar] were included. However, for 21-day-old seedling samples, only aerial parts of the plants grown on soil were included. For *in vivo* confirmation of gene expression experiments, flower stage 14 [76] *ms1* pistils were hand pollinated with wild-type (Col-0) pollen. Pollinated pistils, either 1 minute or 2 hours after pollination, were cut at the junction of the style and ovary and the stigma and style portion (cut pistils) were used for RNA isolation. For unpollinated pistils, flower stage 14 [76] *ms1* cut pistils devoid of any pollen were used. Fifteen cut pistils of each kind were used for each replicate (2) RNA isolation.

### RNA Extraction, Probe Preparation, and Gene Chip Hybridization

Total RNA was extracted from dry pollen, 0.5 h PT, 4 h PT and SIV PT using the Qiagen RNeasy kit (http://www.qiagen.com). The yield and RNA purity were determined by Nano-Drop (Thermo Scientific, Wilmington, DE, USA) and gel electrophoresis. RNA integrity was checked using an Agilent 2100 Bioanalyzer (Agilent Technologies, Boblingen, Germany). Hybridization and post hybridization processing were performed as per the manufacturer's instructions by the Arizona Cancer Center Microarray facility (http://www.azcc.arizona.edu/laboratory/l_microarray.htm). Total RNA (5 µg, dry pollen, 0.5 h PT and 4 h PT) and 2 µg (SIV PT) was processed as per the Affymetrix GeneChip Expression Analysis protocol (Part#701071, Rev 5, Affymetrix, Santa Clara, CA). Briefly, after first and second strand cDNA synthesis with total RNA, the cDNAs were used to generate cRNA labeled with biotin in an *in vitro* transcription reaction. For each pollen condition, labeled cRNA was fragmented and 15 µg of fragmented cRNA (25–200 nt as per Agilent 2100 Bioanalyzer RNA 6000 Nano Chip Series II Assay, Agilent Technologies, Waldbronn, Germany) was hybridized to the GeneChip *Arabidopsis* ATH1 genome arrays (http://www.affymetrix.com) for 20 hours at 45°C. Standard washing and staining procedures were performed using the GeneChip Fluidics Station 450 (Affymetrix, Santa Clara, CA). The arrays were then scanned using the GeneChip Scanner 3000 with 7 G upgrade (Affymetrix, Santa Clara, CA). Signal intensities from each of the 15 arrays were converted to raw expression data (with Present, “P”, Absent, “A” and Marginal, “M” scores) using GeneChip Operating Software (GCOS) (Affymetrix, Santa Clara, CA) and are provided as supplementary files (Table S1, Table S2, Table S3, Table S4).

### Accessing Microarray Data

Raw data (.CEL and CHP files) from all 15 microarrays reported in this study have been deposited in Gene Expression Omnibus [77] public repository and can be accessed from (http://www.ncbi.nlm.nih.gov/geo/query/acc.cgi?acc=GSE17343) using the Series accession number GSE17343.

### Bioinformatic and Statistical Analysis of Affymetrix ATH1 Genome Array Data

In addition to the 15 arrays from this study, we obtained 25 publicly available array data (AtGenExpress, http://www.ebi.ac.uk [28] and stigma and ovary microarray data from Gene Expression Omnibus at http://www.ncbi.nlm.nih.gov/geo/ [29]). The following cel files were downloaded: 7 day old roots–ATGE_3A-C, 17 day old roots–ATGE_9A-C, rosette leaves–ATGE_17A-C, pollen–ATGE_73A-C, 8 day old seedlings–ATGE_96A-C, 21 day old seedlings–ATGE_100A-C. Probe data for ovary–GSM67078.cel, GSM67079.cel, GSM67080.cel, SM67081.cel and stigma–GSM67084.cel, GSM67086.cel, GSM67087.cel.

Using RMA (Robust Microarray Analysis tool in the affy library) [78], we normalized the data from all of these 40 arrays (Table S5). Using the affy and limma BioConductor libraries (http://www.bioconductor.org) and the R programming project (http://www.R-project.org), we calculated the statistical significance in expression level changes of the following comparisons: 0.5 h PT vs. dry pollen; 4 h PT vs. dry pollen; 4 h PT vs. 0.5 h PT; SIV PT vs. dry pollen; SIV PT vs. 4 h PT. After estimating the variance of mean signal intensities for each probe set, the significance of this value in the two conditions was evaluated by performing a t-test [79]. The probabilities obtained were corrected for multiple hypothesis testing by reshuffling the data to obtain an estimate of the false discovery rate (B values) and applying this estimate to lower the probability of the t-value (adjusted P values). The complete results from the statistical analysis for each of the five comparisons and for every probe set in an array are also reported in Table S5.

### Hierarchical and Non-Hierarchical k-Means Clustering

To investigate the relationships among pollen samples, agglomerative hierarchical clustering of the fifteen microarrays representing four pollen conditions, was performed as described [43],[80]. To find genes that had the best discriminative ability, based on its expression profile, we employed non-hierarchical k-means clustering method [43]. For this, we compared dry pollen and 4 h PT hierarchical clusters to the SIV PT hierarchical cluster and in each comparison, for every gene, we calculated discriminative weight, a parameter that measures the ability of a gene's expression values to distinguish two clusters. The discriminative weight of the gene for a pair of clusters is defined by, 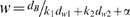
where *d_B_* is the distance between centers of the clusters, *d_wi_* is the average Euclidean distance among all sample pairs within cluster *i*, 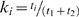 where *t_i_* is the total number of sample pairs in the cluster *i*.

### GO Analysis for SIV PT-Enriched Genes

GO term enrichment analysis reported in Figure 2 was performed essentially as described in [81],[82]. Briefly, the hypergeometric distribution test was applied on the gene sets in sectors 1–4 (Figure 2B) using the GOHyperGAll function [81] which yielded raw and Bonferroni corrected p-values (adjusted p value). GO terms from the “Molecular Function” category that had an adjusted P value of <0.05 were considered highly enriched and are shown in Figures 2C and 2E. *Arabidopsis* gene-to-GO mappings were downloaded from the GO site (10/12/2007 release; http://geneontology.org). A complete list of GO-terms (for all three broad GO categories) associated with sectors 1–4 are provided in Table S7. Within each sector, only unique genes belonging to each GO term category were considered. For the total number of genes for each GO term category reported in Figure 2C, unique numbers of genes from each sector were added, without eliminating gene overlap between sectors. The Fisher exact test was performed to determine if any GO term was significantly overrepresented in SIV PT-enriched genes (Table 1), given that this gene list was smaller compared to those used in Figure 2C [83]. From the SIV PT-enriched gene list (383, Table S8), we excluded genes that were also expressed in pollen samples (ATGE_73A-C); the remaining 357 probes were mapped to genes. Only single probes were chosen if multiple probes mapped to the same gene or a gene family. This criteria resulted in a final list of 349 SIV-enriched genes that was then compared to pollen-expressed genes (ATGE_73A-C, [28] to obtain P-values for overrepresented GO terms in SIV PT-enriched genes. The GO terms with P<0.05 were considered significantly overrepresented in SIV PT-enriched genes and reported in Table 1. Complete results of this analysis and the genes associated with GO-terms listed in Table 1 are provided in Table S9.

### RT-PCR and qRT-PCR

For each of the RT-PCR experiments, new RNA samples were isolated from the indicated cells/tissues, cDNA was synthesized and used as template for PCR (Figure S2) and quantitative real-time PCR (Figure 3 and Table 3). Total RNA was isolated using the Qiagen RNeasy kit followed by treatment with Fermentas DNase I (http://www.fermentas.com) prior to first-strand cDNA synthesis using Invitrogen ThermoScript RT-PCR kit (http://www.invitrogen.com). PCR (with PowerTaq DNA Polymerase PCR system, Altila Biosystems, Palo Alto, CA) was performed as follows: 3 minutes (min) at 94°C, 38 cycles of 30 seconds (sec) at 94°C, 1 min at 60°C and 1 min at 72°C, followed by 5 min at 72°C. Real-time RT-PCR was performed using the Roche FastStart DNA Master SYBR Green I master mix (http://www.roche.com) in a LightCycler system (Roche, http://www.roche.com). The PCR primers used in RT-PCR and qRT-PCR experiments are listed in Table S17. The PCR cycle conditions used for real time PCR were as follows: a 95°C for 5 min followed by 45 cycles of 95°C for 10 sec, 60°C for 15 sec, and 72°C for 15 sec. For each gene analyzed by RT-PCR and qRT-PCR, four reactions were carried out, including two technical replicates and two biological replicates (using RNA from independently harvested tissues). In each qRT-PCR run, ACTIN2 (Threshold Cycle (C_T_) value of 18–19), was used to normalize for mRNA levels. We considered a gene to be expressed only if it had a C_T_ value <36. When expression was not detected in a qRT-PCR reaction, a C_T_ value of 45 (since 45 cycles were used in a real time PCR reaction) was used to calculate the fold change.

### *In Vivo* Pollen Tube Staining

Manually self-pollinated *ms1* pistils were harvested either one minute or 2 hours after pollination. The pollinated pistils were stained with aniline blue to visualize *in vivo* pollen tube growth as described previously [84]. Stained pistils were observed on a Zeiss Axiovert 100 microscope with a Zeiss 365 G filter (Carl Zeiss, Oberkochen, Germany). By this staining procedure, the majority of the pollen tubes reached the style tissue in 2 hours (Figure 4A).

### Reverse Genetic Analysis

SAIL lines were chosen (http://signal.salk.edu/ [85]) with insertions between 300 bp upstream of the 5′ UTR and 300 bp downstream of the 3′UTR (exons were prioritized over introns); and for which there was a TAIL PCR sequence that corroborated the T-DNA insert site to a single locus in the *Arabidopsis* genome. Determination of Basta was performed as reported [49]. Basta plants were transferred to soil and T-DNA insertion sites were confirmed using left border (LB3, LB2, and/or LB1 [86]) and gene-specific ‘right’ primers (Table S16, designed using http://signal.salk.edu/tdnaprimers.2.html) in a PCR reaction. The PCR program used for this reaction was: 94°C for 5 min followed by 36 cycles of 94°C for 15 sec, 60°C for 30 sec, 72°C for 2 min, and a final elongation step of 72°C for 4 min. Pollen tetrads from one stage 14 flower [76] from each Basta plant were stained and assayed for segregation of the LAT52:GUS transgene as described [49]. Transmission of the T-DNA following self-fertilization or through crosses to *ms1* were tested as described [49].

### *In Vitro* Pollen Phenotypic Analysis

Pollen grains were incubated in liquid pollen growth medium [53] on upside-down slides [87] for 6 hours and stained for GUS activity as described [49]. Images were captured (Zeiss Axiovert 200 M, Carl Zeiss, Oberkochen, Germany) and used to determine pollen tube germination rates and pollen tube length for GUS- and GUS+ pollen using ImageJ software (http://rsbweb.nih.gov/ij/docs/faqs.html). These experiments were analyzed as a randomized complete block design with dates of observation as blocks and genotype of pollen (GUS+, insertion; GUS-, wild type) as treatments. Pollen germination and pollen tube length values were subjected to mixed-model analysis of variance with block considered a random effect and treatment a fixed effect. Untransformed least-squares means and P values [88] from this analysis are reported (Table 5). Analysis was done using PROC MIXED in SAS/STAT Version 9.1 of the SAS System for Windows. (Copyright © 2002-2003 SAS Institute Inc).

### *In Vivo* Pollen Phenotypic Analysis

Pollen tube growth in the pistil was examined after crossing pollen from heterozygous insertion plants to three or more *ms1* pistils. Pollinated pistils were harvested 24 hours after pollination, prepared for GUS staining and microscopy observations as described previously [49]. Stained pistils were imaged (differential interference contrast) using a Zeiss Axiovert 200 M microscope (Carl Zeiss, Oberkochen, Germany).

## Supporting Information

Figure S1 Hierarchical clustering of pollen arrays. Agglomerative hierarchical clustering of the fifteen microarrays representing four pollen conditions was performed to generate the dendrogram.
(0.09 MB TIF)

Figures S2 RT-PCR analysis of gene expression. Total RNA from indicated tissues—dry pollen, 0.5 h PT, 4 h PT, 8- and 21-day-old seedlings (DS) —was used as templates to perform oligo-dT primed reverse transcription reactions followed by cDNA synthesis. RT-PCR was performed with cDNAs from indicated tissues and gel images of PCR products amplified are shown. (A) RT-PCR analysis of pollen-enriched and pollen-expressed genes. (B) RT-PCR analysis of genes that are significantly altered in SIV PT compared to 4 h PT. (C) RT-PCR analysis of pistil-dependent gene expression changes in vivo. Samples analyzed were dry pollen, unpollinated ms1 pistils (virgin pistil), ms1 pistils pollinated for one minute (1 m pollinated pistil) and ms1 pistils pollinated for two hours (2 h pollinated pistil).
(1.89 MB TIF)

Figure S3 Heat maps representing microarray data. (A) Genes analyzed by qRT-PCR (Figure 3, Figure 4, and Table 3). Relative expression values obtained from microarray analysis (Table S5) are shown as a heat map. For each gene, the highest value is set at 100% and the relative value is calculated for other samples. (B) Genes chosen for reverse genetic analysis. The top set of genes were chosen because they were significantly higher in 4 h PT compared to dry pollen. The bottom set of genes were chosen because they were significantly higher in SIV PT than in 4 h PT. The heat map displays normalized expression values for all publicly available datasets we analyzed along with our pollen microarray data. The right panel shows a phenotypic summary of our reverse genetic analysis. Male transmission defects were determined by crossing heterozygous insertion pollen to ms1 pistils (Table 4). Germination % and tube length were determined in vitro (Table 5, Figure S4). Ovule targeting was determined by crossing heterozygous insertion pollen to ms1 pistils and counting the number of ovules targeted by insertion pollen tubes (Figure 5).
(0.88 MB TIF)

Figure S4 Five insertions cause defects in pollen tube growth in vitro. Pollen heterozygous for the indicated insertion was grown in vitro for 6 hours and then stained for GUS expression. GUS+ (blue) pollen tubes carry insertions; GUS- (white) pollen tubes are wild type. In control pollen (A), the GUS+ (blue) pollen tubes are as long and as numerous as GUS- (white) pollen tubes (see Table 5 for quantification). Insertions that did not cause severe growth defects are also shown (B–H). Insertions caused severe defects in pollen tube germination (L,N) and/or in tube length (I–P). Higher magnification images of indicated insertions are shown (N–P). Arrowheads point to representative GUS+ (black arrowhead) and GUS- (white arrowhead) in (A); all GUS+ pollen tubes are highlighted with black arrowheads in (O) and (P). Scale bars = 100 µm.
(1.54 MB TIF)

Figure S5 Members of subgroup 18 are expressed in growing pollen tubes. Mean microarray expression data (log2, Table S5) are plotted for each of the cell or tissue types analyzed in this study for seven genes that comprise MYB subgroup 18 [60],[61].
(0.25 MB TIF)

Table S1 Raw gene expression data for dry pollen
(6.27 MB XLS)

Table S2 Raw gene expression data for 0.5 h PT
(6.27 MB XLS)

Table S3 Raw gene expression data for 4 h PT
(6.27 MB XLS)

Table S4 Raw gene expression data for SIV PT. Normalized expression data (log2) for each replicate of dry pollen, 0.5 h PT, 4 h PT, SIV PT, and 25 selected experiments from publicly available sources; and statistical analyses for selected comparisons.
(5.95 MB XLS)

Table S5 Normalized expression data (log2) for each replicate of dry pollen, 0.5 h PT, 4 h PT, SIV PT, and 25 selected experiments from publicly available sources; and statistical analyses for selected comparisons. Statistical analyses of significant differences between two indicated conditions are described in Materials and Methods. The table has been sorted by Affymetrix ID (ascending). FC = Fold Change; B value = False discovery rate. Affymetrix probe sets (ID, Column A) associated with a single nuclear gene are identified by a Genbank number (column D) that begins At1G, At2G, At3G, At4G, or At5G; mitochondrial genes begin AtMG, chloroplast genes begin AtCG. Affymetrix probe sets associated with multiple genes are identified as ‘multiple’ in column D. Affymetrix probe sets that are not associated with genes are blank in column D. Genes associated with multiple Affymetrix probe sets are flagged (*) in column E. Affymetrix control probe sets begin ‘AFFX-’ (Column A), these were not counted in analyses.
(6.81 MB ZIP)

Table S6 Pearson correlation coefficients between microarray experiments. The Pearson correlation coefficients between quantile normalized 40 microarray data sets used in this study are shown.
(0.04 MB XLS)

Table S7 Complete list of overrepresented GO terms shown in Figure 2C and Figure 2E
(2.25 MB XLS)

Table S8 SIV PT-enriched genes
(0.51 MB XLS)

Table S9 Complete list of overrepresented GO terms and the associated genes from the Fisher exact test shown in Table 1
(0.17 MB XLS)

Table S10 Statistical analysis of significant gene expression changes between 0.5 h PT and dry pollen
(0.05 MB XLS)

Table S11 Statistical analysis of gene expression changes between 4 h PT and 0.5 h PT
(0.15 MB XLS)

Table S12 Statistical analysis of gene expression changes between 4 h PT and dry pollen
(0.21 MB XLS)

Table S13 Statistical analysis of gene expression changes between SIV PT and dry pollen
(1.36 MB XLS)

Table S14 Statistical analysis of gene expression changes between SIV PT v 4 h PT
(0.99 MB XLS)

Table S15 Genes that best discriminate the SIV PT cluster from other pollen clusters
(0.06 MB DOC)

Table S16 Syngenta Arabidopsis Insertion Lines (SAIL) analyzed
(0.04 MB XLS)

Table S17 List of primers used in RT-PCR and qRT-PCR experiments
(0.02 MB XLS)

## References

[1] Bishopp A, Mahonen AP, Helariutta Y (2006). Signs of change: hormone receptors that regulate plant development.. Development.

[2] Yadegari R, Drews GN (2004). Female gametophyte development.. Plant Cell.

[3] Borg M, Brownfield L, Twell D (2009). Male gametophyte development: a molecular perspective.. J Exp Bot.

[4] McCormick S (2004). Control of male gametophyte development.. Plant Cell.

[5] von Besser K, Frank AC, Johnson MA, Preuss D (2006). Arabidopsis HAP2 (GCS1) is a sperm-specific gene required for pollen tube guidance and fertilization.. Development.

[6] Yang Z (2008). Cell polarity signaling in Arabidopsis.. Annu Rev Cell Dev Biol.

[7] Cardenas L, Lovy-Wheeler A, Kunkel JG, Hepler PK (2008). Pollen tube growth oscillations and intracellular calcium levels are reversibly modulated by actin polymerization.. Plant Physiol.

[8] Cheung AY, Wu HM (2008). Structural and signaling networks for the polar cell growth machinery in pollen tubes.. Annu Rev Plant Biol.

[9] Geitmann A, Palanivelu R (2007). Fertilization Requires Communication: Signal Generation and Perception During Pollen Tube Guidance.. Floriculture and Ornamental Biotechnology.

[10] Okuda S, Tsutsui H, Shiina K, Sprunck S, Takeuchi H (2009). Defensin-like polypeptide LUREs are pollen tube attractants secreted from synergid cells.. Nature.

[11] Sandaklie-Nikolova L, Palanivelu R, King EJ, Copenhaver GP, Drews GN (2007). Synergid cell death in Arabidopsis is triggered following direct interaction with the pollen tube.. Plant Physiol.

[12] Escobar-Restrepo JM, Huck N, Kessler S, Gagliardini V, Gheyselinck J (2007). The FERONIA receptor-like kinase mediates male-female interactions during pollen tube reception.. Science.

[13] Capron A, Gourgues M, Neiva LS, Faure JE, Berger F (2008). Maternal control of male-gamete delivery in Arabidopsis involves a putative GPI-anchored protein encoded by the LORELEI gene.. Plant Cell.

[14] Christensen CA, Gorsich SW, Brown RH, Jones LG, Brown J (2002). Mitochondrial GFA2 is required for synergid cell death in Arabidopsis.. Plant Cell.

[15] Ingouff M, Hamamura Y, Gourgues M, Higashiyama T, Berger F (2007). Distinct dynamics of HISTONE3 variants between the two fertilization products in plants.. Curr Biol.

[16] Becker JD, Boavida LC, Carneiro J, Haury M, Feijo JA (2003). Transcriptional profiling of Arabidopsis tissues reveals the unique characteristics of the pollen transcriptome.. Plant Physiology.

[17] Honys D, Twell D (2003). Comparative analysis of the Arabidopsis pollen transcriptome.. Plant Physiol.

[18] Honys D, Twell D (2004). Transcriptome analysis of haploid male gametophyte development in Arabidopsis.. Genome Biol.

[19] Pina C, Pinto F, Feijo JA, Becker JD (2005). Gene family analysis of the Arabidopsis pollen transcriptome reveals biological implications for cell growth, division control, and gene expression regulation.. Plant Physiology.

[20] Borges F, Gomes G, Gardner R, Moreno N, McCormick S (2008). Comparative transcriptomics of Arabidopsis sperm cells.. Plant Physiol.

[21] Wang Y, Zhang WZ, Song LF, Zou JJ, Su Z (2008). Transcriptome analyses show changes in gene expression to accompany pollen germination and tube growth in Arabidopsis.. Plant Physiol.

[22] Mo Y, Nagel C, Taylor LP (1992). Biochemical complementation of chalcone synthase mutants defines a role for flavonols in functional pollen.. Proc Natl Acad Sci U S A.

[23] Guyon VN, Astwood JD, Garner EC, Dunker AK, Taylor LP (2000). Isolation and characterization of cDNAs expressed in the early stages of flavonol-induced pollen germination in petunia.. Plant Physiol.

[24] Taylor LP, Hepler PK (1997). Pollen Germination and Tube Growth.. Annu Rev Plant Physiol Plant Mol Biol.

[25] Higashiyama T, Kuroiwa H, Kawano S, Kuroiwa T (1998). Guidance in vitro of the pollen tube to the naked embryo sac of torenia fournieri.. Plant Cell.

[26] Palanivelu R, Preuss D (2006). Distinct short-range ovule signals attract or repel Arabidopsis thaliana pollen tubes in vitro.. BMC Plant Biol.

[27] Meyers BC, Kozik A, Griego A, Kuang H, Michelmore RW (2003). Genome-wide analysis of NBS-LRR-encoding genes in Arabidopsis.. Plant Cell.

[28] Schmid M, Davison TS, Henz SR, Pape UJ, Demar M (2005). A gene expression map of Arabidopsis thaliana development.. Nat Genet.

[29] Swanson R, Clark T, Preuss D (2005). Expression profiling of Arabidopsis stigma tissue identifies stigma-specific genes.. Sexual Plant Reproduction.

[30] Li H, Wu G, Ware D, Davis KR, Yang Z (1998). Arabidopsis Rho-related GTPases: differential gene expression in pollen and polar localization in fission yeast.. Plant Physiol.

[31] Zhang Y, McCormick S (2007). A distinct mechanism regulating a pollen-specific guanine nucleotide exchange factor for the small GTPase Rop in Arabidopsis thaliana.. Proc Natl Acad Sci U S A.

[32] Szumlanski AL, Nielsen E (2009). The Rab GTPase RabA4d Regulates Pollen Tube Tip Growth in Arabidopsis thaliana.. Plant Cell.

[33] Schiott M, Romanowsky SM, Baekgaard L, Jakobsen MK, Palmgren MG (2004). A plant plasma membrane Ca2+ pump is required for normal pollen tube growth and fertilization.. Proc Natl Acad Sci U S A.

[34] Frietsch S, Wang YF, Sladek C, Poulsen LR, Romanowsky SM (2007). A cyclic nucleotide-gated channel is essential for polarized tip growth of pollen.. Proc Natl Acad Sci U S A.

[35] Jiang L, Yang SL, Xie LF, Puah CS, Zhang XQ (2005). VANGUARD1 encodes a pectin methylesterase that enhances pollen tube growth in the Arabidopsis style and transmitting tract.. Plant Cell.

[36] Kobayashi K, Awai K, Takamiya K, Ohta H (2004). Arabidopsis type B monogalactosyldiacylglycerol synthase genes are expressed during pollen tube growth and induced by phosphate starvation.. Plant Physiol.

[37] Huang L, Cao J, Zhang A, Ye Y, Zhang Y (2009). The polygalacturonase gene BcMF2 from Brassica campestris is associated with intine development.. J Exp Bot.

[38] Parre E, Geitmann A (2005). Pectin and the role of the physical properties of the cell wall in pollen tube growth of Solanum chacoense.. Planta.

[39] Bock KW, Honys D, Ward JM, Padmanaban S, Nawrocki EP (2006). Integrating membrane transport with male gametophyte development and function through transcriptomics.. Plant Physiol.

[40] Stadler R, Truernit E, Gahrtz M, Sauer N (1999). The AtSUC1 sucrose carrier may represent the osmotic driving force for anther dehiscence and pollen tube growth in Arabidopsis.. Plant J.

[41] Hepler PK, Vidali L, Cheung AY (2001). Polarized cell growth in higher plants.. Annu Rev Cell Dev Biol.

[42] Holdaway-Clarke TL, Weddle NM, Kim S, Robi A, Parris C (2003). Effect of extracellular calcium, pH and borate on growth oscillations in Lilium formosanum pollen tubes.. J Exp Bot.

[43] Bittner M, Meltzer P, Chen Y, Jiang Y, Seftor E (2000). Molecular classification of cutaneous malignant melanoma by gene expression profiling.. Nature.

[44] Franklin-Tong VE (1999). Signaling and the modulation of pollen tube growth.. Plant Cell.

[45] Bosch M, Cheung AY, Hepler PK (2005). Pectin methylesterase, a regulator of pollen tube growth.. Plant Physiol.

[46] Sessions A, Burke E, Presting G, Aux G, McElver J (2002). A high-throughput Arabidopsis reverse genetics system.. Plant Cell.

[47] Twell D, Wing R, Yamaguchi J, McCormick S (1989). Isolation and expression of an anther-specific gene from tomato.. Mol Gen Genet.

[48] Preuss D, Rhee SY, Davis RW (1994). Tetrad analysis possible in Arabidopsis with mutation of the *QUARTET (QRT)* genes.. Science.

[49] Johnson MA, von Besser K, Zhou Q, Smith E, Aux G (2004). Arabidopsis hapless mutations define essential gametophytic functions.. Genetics.

[50] Drews GN, Yadegari R (2002). Development and function of the angiosperm female gametophyte.. Annu Rev Genet.

[51] Chen J, Deng F, Li J, Wang QJ (2008). Selective binding of phorbol esters and diacylglycerol by individual C1 domains of the PKD family.. Biochem J.

[52] Cruz-Ramirez A, Lopez-Bucio J, Ramirez-Pimentel G, Zurita-Silva A, Sanchez-Calderon L (2004). The xipotl mutant of Arabidopsis reveals a critical role for phospholipid metabolism in root system development and epidermal cell integrity.. Plant Cell.

[53] Boavida LC, McCormick S (2007). Temperature as a determinant factor for increased and reproducible in vitro pollen germination in Arabidopsis thaliana.. Plant J.

[54] Mollet JC, Park SY, Nothnagel EA, Lord EM (2000). A lily stylar pectin is necessary for pollen tube adhesion to an in vitro stylar matrix.. Plant Cell.

[55] Kim S, Mollet JC, Dong J, Zhang K, Park SY (2003). Chemocyanin, a small basic protein from the lily stigma, induces pollen tube chemotropism.. Proc Natl Acad Sci U S A.

[56] Burch-Smith TM, Dinesh-Kumar SP (2007). The functions of plant TIR domains.. Sci STKE.

[57] Faigon-Soverna A, Harmon FG, Storani L, Karayekov E, Staneloni RJ (2006). A constitutive shade-avoidance mutant implicates TIR-NBS-LRR proteins in Arabidopsis photomorphogenic development.. Plant Cell.

[58] Swanson WJ, Vacquier VD (2002). The rapid evolution of reproductive proteins.. Nat Rev Genet.

[59] Swanson WJ, Wong A, Wolfner MF, Aquadro CF (2004). Evolutionary expressed sequence tag analysis of Drosophila female reproductive tracts identifies genes subjected to positive selection.. Genetics.

[60] Friedman WE (1999). Expression of the cell cycle in sperm of Arabidopsis: implications for understanding patterns of gametogenesis and fertilization in plants and other eukaryotes.. Development.

[61] Procissi A, Guyon A, Pierson ES, Giritch A, Knuiman B (2003). KINKY POLLEN encodes a SABRE-like protein required for tip growth in Arabidopsis and conserved among eukaryotes.. Plant J.

[62] Kranz HD, Denekamp M, Greco R, Jin H, Leyva A (1998). Towards functional characterisation of the members of the R2R3-MYB gene family from Arabidopsis thaliana.. Plant J.

[63] Stracke R, Werber M, Weisshaar B (2001). The R2R3-MYB gene family in Arabidopsis thaliana.. Curr Opin Plant Biol.

[64] Schwager KM, Calderon-Villalobos LI, Dohmann EM, Willige BC, Knierer S (2007). Characterization of the VIER F-BOX PROTEINE genes from Arabidopsis reveals their importance for plant growth and development.. Plant Cell.

[65] Gusti A, Baumberger N, Nowack M, Pusch S, Eisler H (2009). The Arabidopsis thaliana F-box protein FBL17 is essential for progression through the second mitosis during pollen development.. PLoS ONE.

[66] Kim HJ, Oh SA, Brownfield L, Hong SH, Ryu H (2008). Control of plant germline proliferation by SCF(FBL17) degradation of cell cycle inhibitors.. Nature.

[67] Kepinski S, Leyser O (2005). The Arabidopsis F-box protein TIR1 is an auxin receptor.. Nature.

[68] Dharmasiri N, Dharmasiri S, Estelle M (2005). The F-box protein TIR1 is an auxin receptor.. Nature.

[69] Zhang D, Wengier D, Shuai B, Gui CP, Muschietti J (2008). The pollen receptor kinase LePRK2 mediates growth-promoting signals and positively regulates pollen germination and tube growth.. Plant Physiol.

[70] Muschietti J, Eyal Y, McCormick S (1998). Pollen tube localization implies a role in pollen-pistil interactions for the tomato receptor-like protein kinases LePRK1 and LePRK2.. Plant Cell.

[71] Zou J, Song L, Zhang W, Wang Y, Ruan S (2009). Comparative proteomic analysis of Arabidopsis mature pollen and germinated pollen.. J Integr Plant Biol.

[72] Holmes-Davis R, Tanaka CK, Vensel WH, Hurkman WJ, McCormick S (2005). Proteome mapping of mature pollen of Arabidopsis thaliana.. Proteomics.

[73] Grobei MA, Qeli E, Brunner E, Rehrauer H, Zhang R (2009). Deterministic protein inference for shotgun proteomics data provides new insights into Arabidopsis pollen development and function.. Genome Res.

[74] Johnson-Brousseau SA, McCormick S (2004). A compendium of methods useful for characterizing Arabidopsis pollen mutants and gametophytically-expressed genes.. Plant J.

[75] Palanivelu R, Brass L, Edlund AF, Preuss D (2003). Pollen tube growth and guidance is regulated by POP2, an Arabidopsis gene that controls GABA levels.. Cell.

[76] Smyth DR, Bowman JL, Meyerowitz EM (1990). Early flower development in Arabidopsis.. Plant Cell.

[77] Edgar R, Domrachev M, Lash AE (2002). Gene Expression Omnibus: NCBI gene expression and hybridization array data repository.. Nucleic Acids Res.

[78] Irizarry RA, Hobbs B, Collin F, Beazer-Barclay YD, Antonellis KJ (2003). Exploration, normalization, and summaries of high density oligonucleotide array probe level data.. Biostatistics.

[79] Smyth GK (2004). Linear models and empirical Bayes methods for assessing differential expression in microarray experiments.. Stat Appl Genet Mol Biol.

[80] Everitt B (1992). Applied Multivariate Data Analysis..

[81] Horan K, Jang C, Bailey-Serres J, Mittler R, Shelton C (2008). Annotating genes of known and unknown function by large-scale coexpression analysis.. Plant Physiol.

[82] Yadav RK, Girke T, Pasala S, Xie M, Reddy GV (2009). Gene expression map of the Arabidopsis shoot apical meristem stem cell niche.. Proc Natl Acad Sci U S A.

[83] Tanner SW, Agarwal P (2008). Gene Vector Analysis (Geneva): a unified method to detect differentially-regulated gene sets and similar microarray experiments.. BMC Bioinformatics.

[84] Preuss D, Lemieux B, Yen G, Davis RW (1993). A conditional sterile mutation eliminates surface components from Arabidopsis pollen and disrupts cell signaling during fertilization.. Genes Dev.

[85] Alonso JM, Stepanova AN, Leisse TJ, Kim CJ, Chen H (2003). Genome-wide insertional mutagenesis of Arabidopsis thaliana.. Science.

[86] McElver J, Tzafrir I, Aux G, Rogers R, Ashby C (2001). Insertional mutagenesis of genes required for seed development in Arabidopsis thaliana.. Genetics.

[87] Hicks GR, Rojo E, Hong S, Carter DG, Raikhel NV (2004). Geminating pollen has tubular vacuoles, displays highly dynamic vacuole biogenesis, and requires VACUOLESS1 for proper function.. Plant Physiol.

[88] Searle S, Speed FM, Milliken GA (1980). Population marginal means in the linear model: an alternative to least squares means.. American Statistician.

