# Correction: Penetration of the Stigma and Style Elicits a Novel Transcriptome in Pollen Tubes, Pointing to Genes Critical for Growth in a Pistil

**DOI:** 10.1371/journal.pgen.1006210

**Published:** 2016-07-19

**Authors:** Yuan Qin, Alexander R. Leydon, Ann Manziello, Ritu Pandey, David Mount, Stojan Denic, Bane Vasic, Mark A. Johnson, Ravishankar Palanivelu

Concerns were raised regarding errors in panel B of S2 Fig. Specifically, the RT-PCR gel electrophoresis results were duplicated for At3g06830 and At1g73630, and for At3g01820 and At2g28080. The authors determined the source of the errors that were made in the preparation of S2 Fig and have provided a corrected figure here.

## Supporting Information

S2 Fig RT-PCR analysis of gene expression. Total RNA from indicated tissues—dry pollen, 0.5 h PT, 4 h PT, 8- and 21-day-old seedlings (DS)—was used as templates to perform oligo-dT primed reverse transcription reactions followed by cDNA synthesis. RT-PCR was performed with cDNAs from indicated tissues and gel images of PCR products amplified are shown. (A) RT-PCR analysis of pollen-enriched and pollen-expressed genes. (B) RT-PCR analysis of genes that are significantly altered in SIV PT compared to 4 h PT. (C) RT-PCR analysis of pistil-dependent gene expression changes in vivo. Samples analyzed were dry pollen, unpollinated ms1 pistils (virgin pistil), ms1 pistils pollinated for one minute (1 m pollinated pistil) and ms1 pistils pollinated for two hours (2 h pollinated pistil).
(TIF)

## References

[1] Qin Y, Leydon AR, Manziello A, Pandey R, Mount D, Denic S, et al (2009) Penetration of the Stigma and Style Elicits a Novel Transcriptome in Pollen Tubes, Pointing to Genes Critical for Growth in a Pistil. PLoS Genet 5(8): e1000621 doi: 10.1371/journal.pgen.1000621 19714218 10.1371/journal.pgen.1000621 PMC2726614

