# Supplementary figures and images for: Penetration of the Stigma and Style Elicits a Novel Transcriptome in Pollen Tubes, Pointing to Genes Critical for Growth in a Pistil
Source: PLoS Genet. 2009 Aug 28;5(8):e1000621. doi: 10.1371/journal.pgen.1000621 (PMC2726614; doi:10.1371/journal.pgen.1000621)

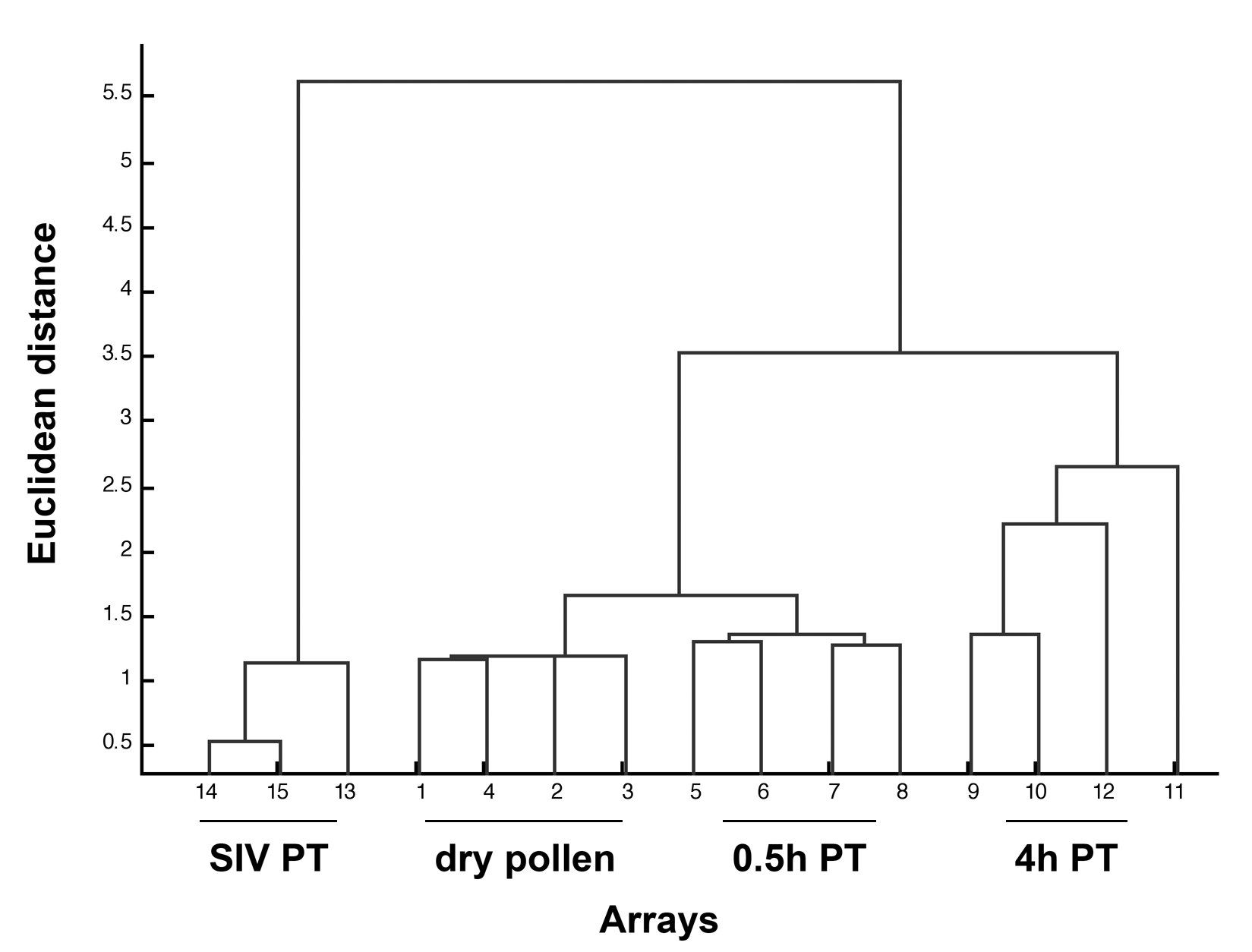

Supplement: Figure S1 — Hierarchical clustering of pollen arrays. Agglomerative hierarchical clustering of the fifteen microarrays representing four pollen conditions was performed to generate the dendrogram. (0.09 MB TIF) [file pgen.1000621.s001.tif]

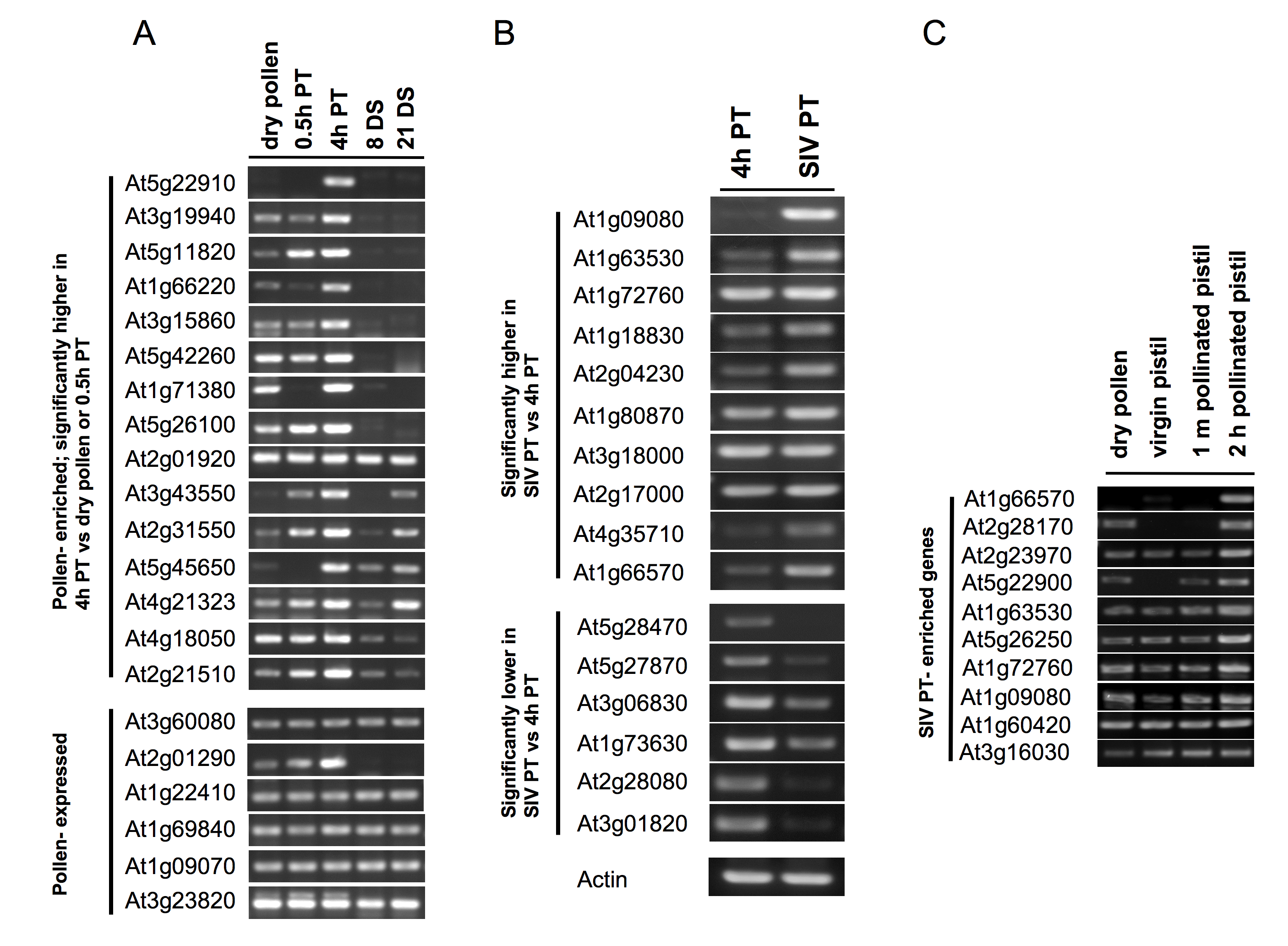

Supplement: Figures S2 — RT-PCR analysis of gene expression. Total RNA from indicated tissues—dry pollen, 0.5 h PT, 4 h PT, 8- and 21-day-old seedlings (DS) —was used as templates to perform oligo-dT primed reverse transcription reactions followed by cDNA synthesis. RT-PCR was performed with cDNAs from indicated tissues and gel images of PCR products amplified are shown. (A) RT-PCR analysis of pollen-enriched and pollen-expressed genes. (B) RT-PCR analysis of genes that are significantly altered in SIV PT compared to 4 h PT. (C) RT-PCR analysis of pistil-dependent gene expression changes in vivo. Samples analyzed were dry pollen, unpollinated ms1 pistils (virgin pistil), ms1 pistils pollinated for one minute (1 m pollinated pistil) and ms1 pistils pollinated for two hours (2 h pollinated pistil). (1.89 MB TIF) [file pgen.1000621.s002.tif]

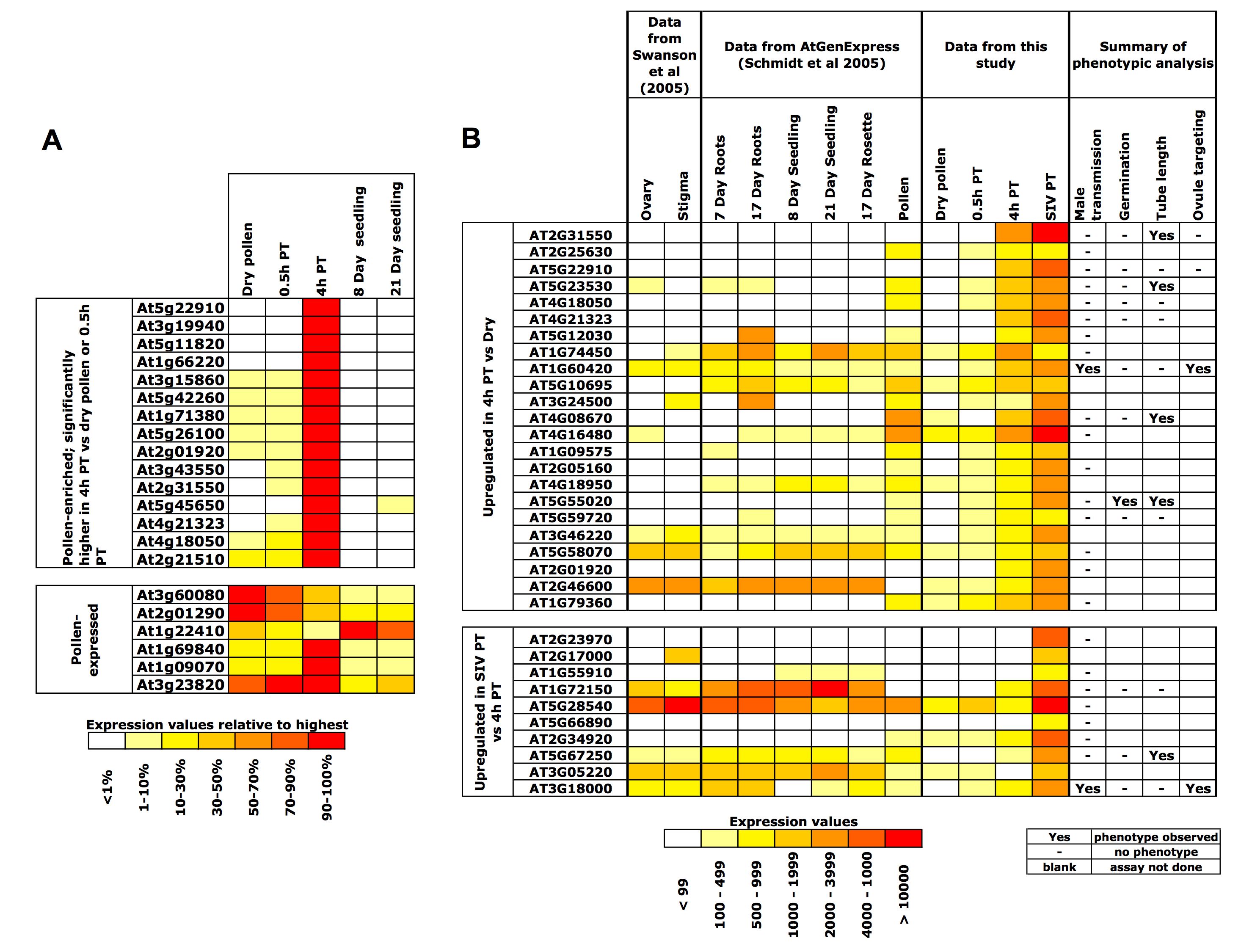

Supplement: Figure S3 — Heat maps representing microarray data. (A) Genes analyzed by qRT-PCR (Figure 3, Figure 4, and Table 3). Relative expression values obtained from microarray analysis (Table S5) are shown as a heat map. For each gene, the highest value is set at 100% and the relative value is calculated for other samples. (B) Genes chosen for reverse genetic analysis. The top set of genes were chosen because they were significantly higher in 4 h PT compared to dry pollen. The bottom set of genes were chosen because they were significantly higher in SIV PT than in 4 h PT. The heat map displays normalized expression values for all publicly available datasets we analyzed along with our pollen microarray data. The right panel shows a phenotypic summary of our reverse genetic analysis. Male transmission defects were determined by crossing heterozygous insertion pollen to ms1 pistils (Table 4). Germination % and tube length were determined in vitro (Table 5, Figure S4). Ovule targeting was determined by crossing heterozygous insertion pollen to ms1 pistils and counting the number of ovules targeted by insertion pollen tubes (Figure 5). (0.88 MB TIF) [file pgen.1000621.s003.tif]

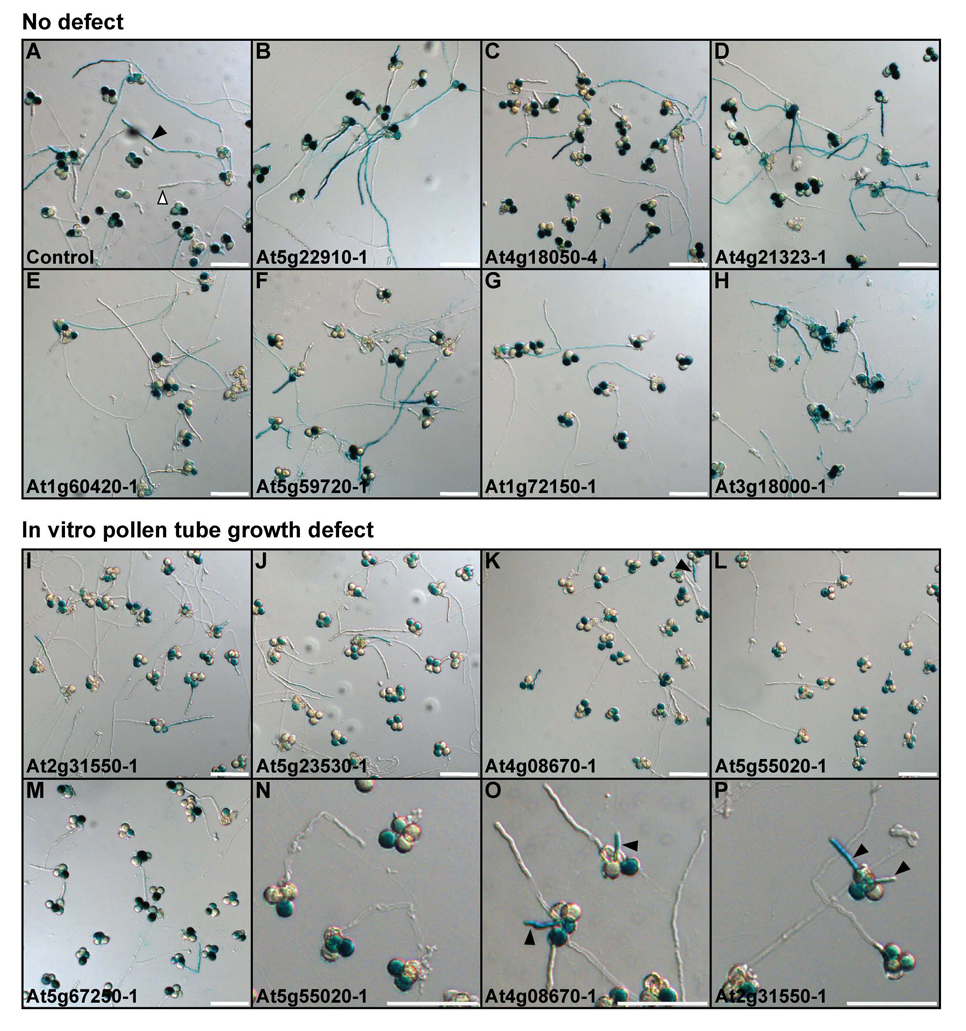

Supplement: Figure S4 — Five insertions cause defects in pollen tube growth in vitro. Pollen heterozygous for the indicated insertion was grown in vitro for 6 hours and then stained for GUS expression. GUS+ (blue) pollen tubes carry insertions; GUS- (white) pollen tubes are wild type. In control pollen (A), the GUS+ (blue) pollen tubes are as long and as numerous as GUS- (white) pollen tubes (see Table 5 for quantification). Insertions that did not cause severe growth defects are also shown (B–H). Insertions caused severe defects in pollen tube germination (L,N) and/or in tube length (I–P). Higher magnification images of indicated insertions are shown (N–P). Arrowheads point to representative GUS+ (black arrowhead) and GUS- (white arrowhead) in (A); all GUS+ pollen tubes are highlighted with black arrowheads in (O) and (P). Scale bars = 100 µm. (1.54 MB TIF) [file pgen.1000621.s004.tif]

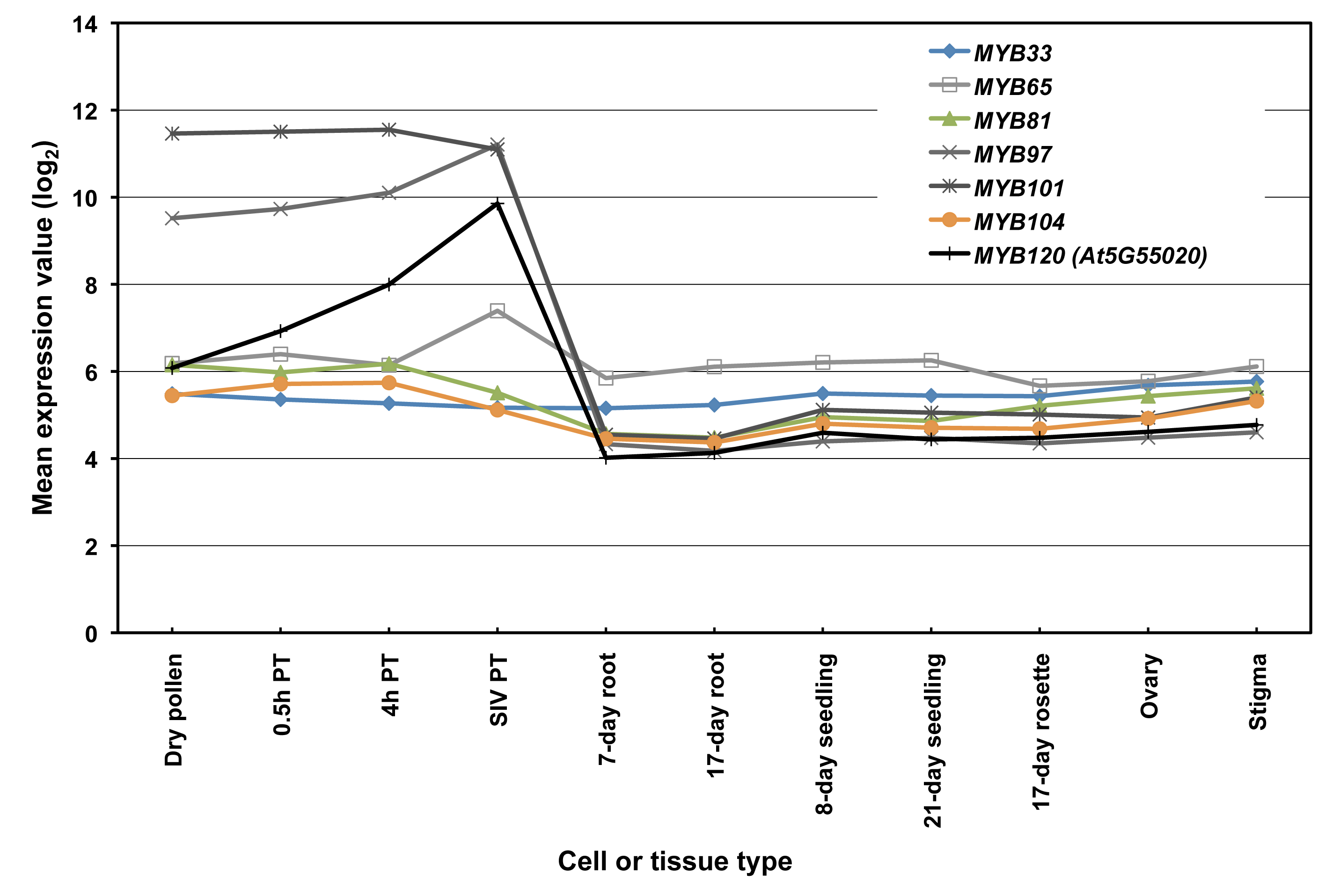

Supplement: Figure S5 — Members of subgroup 18 are expressed in growing pollen tubes. Mean microarray expression data (log2, Table S5) are plotted for each of the cell or tissue types analyzed in this study for seven genes that comprise MYB subgroup 18 [60],[61]. (0.25 MB TIF) [file pgen.1000621.s005.tif]
