# Supplementary material for: Penetration of the Stigma and Style Elicits a Novel Transcriptome in Pollen Tubes, Pointing to Genes Critical for Growth in a Pistil
Source: PLoS Genet. 2009 Aug 28;5(8):e1000621. doi: 10.1371/journal.pgen.1000621 (PMC2726614; doi:10.1371/journal.pgen.1000621)
Supplement: Table S15 — Genes that best discriminate the SIV PT cluster from other pollen clusters (0.06 MB DOC) [file pgen.1000621.s020.doc]

| **Table S15.** Genes that best discriminate the SIV PT cluster from other pollen clusters | | | |
| --- | --- | --- | --- |
| Probe ID | GenBank ID | Gene description | Discriminative weights |
| SIV PT v dry pollen | | | |
| 261545_at | At1g63530 | similar to hydyroxyproline-rich glycoprotein | 10.03 |
| 256848_at | At3g27960 | kinesin light chain-related protein | 9.940 |
| 259803_at | At1g72150 | novel cell-plate-associated protein | 9.856 |
| 253246_at | At4g34600 | unknown protein | 9.836 |
| 254403_at | At4g21323 | subtilase family protein; | 9.834 |
| 258103_at | At3g23630 | isopentenyl transferase involved in cytokinin biosynthesis | 9.830 |
| 246136_at | At5g28470 | transporter | 9.704 |
| 266492_at | At2g07020 | protein kinase family protein | 9.700 |
| 246839_at | At5g26720 | unknown protein | 9.668 |
| 250535_at | At5g08480 | VQ motif-containing protein | 9.635 |
| SIV PT v 4h PT | | | |
| 258739_at | multiple | unknown protein | 6.179 |
| 264648_at | At1g09080 | luminal binding protein 3 | 5.996 |
| 263397_at | multiple | GDSL-motif lipase/hydrolase | 5.910 |
| 249794_at | At5g23530 | Similar to esterase/lipase/thioesterase | 5.908 |
| 259962_at | At1g53690 | DNA-directed RNA polymerase I, II and III 7kDa subunit | 5.896 |
| 265904_at | At2g25630 | glycosyl hydrolase | 5.859 |
| 246136_at | At5g28470 | transporter | 5.858 |
| 267169_at | At2g37540 | short chain-dehydrogenase/reductase | 5.854 |
| 258279_at | At3g26870 | Self-incompatibility-related protein | 5.820 |
| 263169_at | At1g03010 | Phototropic-responsive NPH3 family protein | 5.812 |
