# Supplementary figures and images for: Correction: Penetration of the Stigma and Style Elicits a Novel Transcriptome in Pollen Tubes, Pointing to Genes Critical for Growth in a Pistil
Source: PLoS Genet. 2016 Jul 19;12(7):e1006210. doi: 10.1371/journal.pgen.1006210 (PMC4951096; doi:10.1371/journal.pgen.1006210)

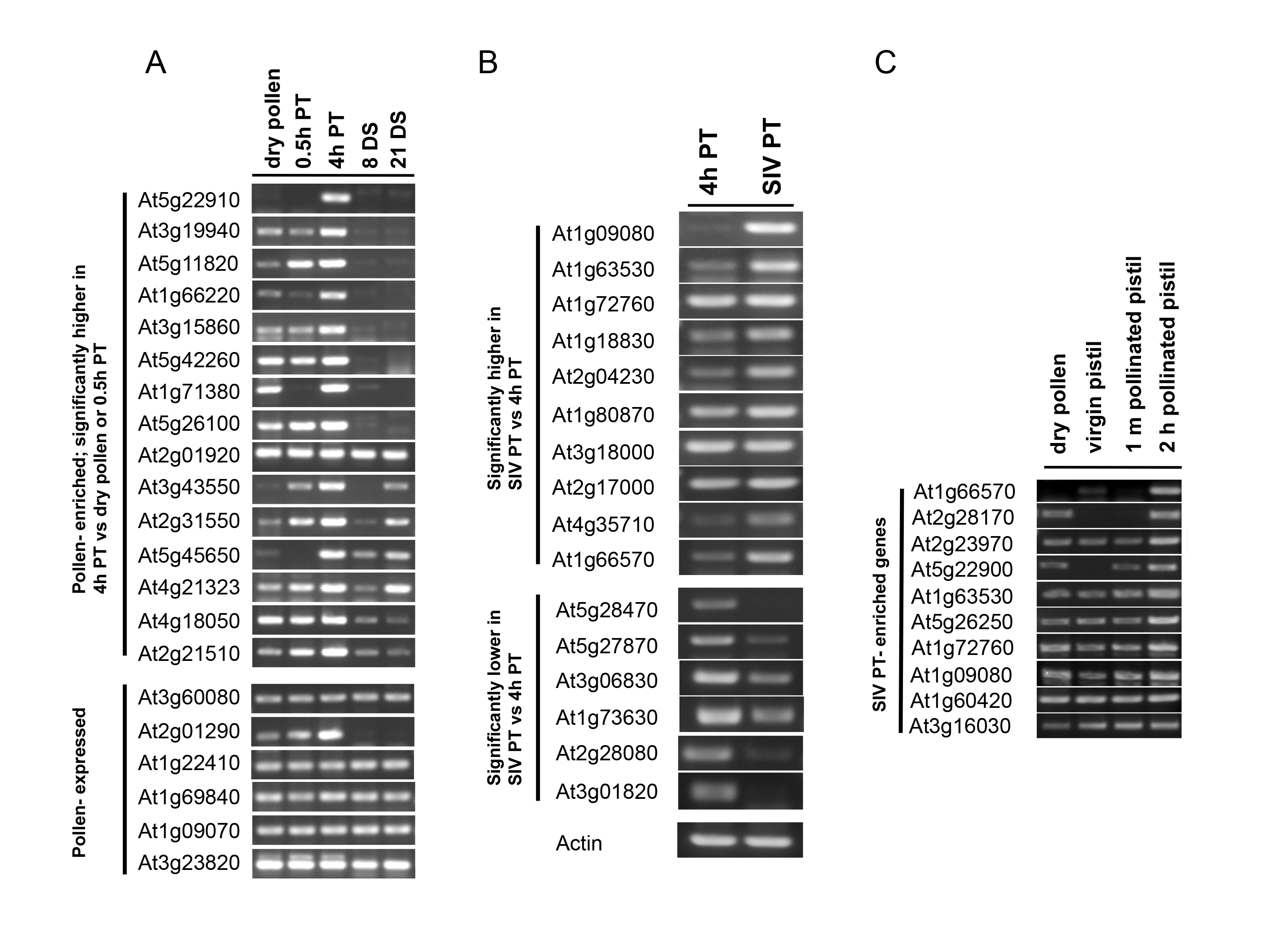

Supplement: S2 Fig — Total RNA from indicated tissues—dry pollen, 0.5 h PT, 4 h PT, 8- and 21-day-old seedlings (DS)—was used as templates to perform oligo-dT primed reverse transcription reactions followed by cDNA synthesis. RT-PCR was performed with cDNAs from indicated tissues and gel images of PCR products amplified are shown. (A) RT-PCR analysis of pollen-enriched and pollen-expressed genes. (B) RT-PCR analysis of genes that are significantly altered in SIV PT compared to 4 h PT. (C) RT-PCR analysis of pistil-dependent gene expression changes in vivo. Samples analyzed were dry pollen, unpollinated ms1 pistils (virgin pistil), ms1 pistils pollinated for one minute (1 m pollinated pistil) and ms1 pistils pollinated for two hours (2 h pollinated pistil). (TIF) [file pgen.1006210.s001.tif]
